# Generation of contractile forces by three-dimensional bundled axonal tracts in micro-tissue engineered neural networks

**DOI:** 10.3389/fnmol.2024.1346696

**Published:** 2024-03-25

**Authors:** Dimple Chouhan, Wisberty J. Gordián Vélez, Laura A. Struzyna, Dayo O. Adewole, Erin R. Cullen, Justin C. Burrell, John C. O’Donnell, D. Kacy Cullen

**Affiliations:** ^1^Department of Neurosurgery, Center for Brain Injury and Repair, Perelman School of Medicine, University of Pennsylvania, Philadelphia, PA, United States; ^2^Center for Neurotrauma, Neurodegeneration and Restoration, Michael J. Crescenz Veterans Affairs Medical Center, Philadelphia, PA, United States; ^3^Department of Bioengineering, School of Engineering and Applied Science, University of Pennsylvania, Philadelphia, PA, United States

**Keywords:** axon tracts, mechanical forces, axon contraction, axon mechanics, cortical neurons

## Abstract

Axonal extension and retraction are ongoing processes that occur throughout all developmental stages of an organism. The ability of axons to produce mechanical forces internally and respond to externally generated forces is crucial for nervous system development, maintenance, and plasticity. Such axonal mechanobiological phenomena have typically been evaluated *in vitro* at a single-cell level, but these mechanisms have not been studied when axons are present in a bundled three-dimensional (3D) form like in native tissue. In an attempt to emulate native cortico-cortical interactions under *in vitro* conditions, we present our approach to utilize previously described micro-tissue engineered neural networks (micro-TENNs). Here, micro-TENNs were comprised of discrete populations of rat cortical neurons that were spanned by 3D bundled axonal tracts and physically integrated with each other. We found that these bundled axonal tracts inherently exhibited an ability to generate contractile forces as the microtissue matured. We therefore utilized this micro-TENN testbed to characterize the intrinsic contractile forces generated by the integrated axonal tracts in the absence of any external force. We found that contractile forces generated by bundled axons were dependent on microtubule stability. Moreover, these intra-axonal contractile forces could simultaneously generate tensile forces to induce so-called axonal “stretch-growth” in different axonal tracts within the same microtissue. The culmination of axonal contraction generally occurred with the fusion of both the neuronal somatic regions along the axonal tracts, therefore perhaps showing the innate tendency of cortical neurons to minimize their wiring distance, a phenomenon also perceived during brain morphogenesis. In future applications, this testbed may be used to investigate mechanisms of neuroanatomical development and those underlying certain neurodevelopmental disorders.

## 1 Introduction

Nervous system development comprises complex dynamic processes that include a multitude of biochemical and physical cues (Van Essen, 1997, 2020; Abuwarda and Pathak, 2020). The role of mechanical forces in driving biological processes during central nervous system (CNS) and peripheral nervous system (PNS) development has recently gained significant attention. Indeed, there is increasing appreciation for the critical role of force generation and mechanosensation in neuronal migration, axonal extension, neurotransmission, and neuronal network development (Anava et al., 2009; Siechen et al., 2009; Smith, 2009; Ayali, 2010; Hanein et al., 2011; Keating and Cullen, 2021). However, the sources of various mechanical forces – as well as the interplay between intrinsic and extrinsic mechano-chemical factors – that collectively lead to the formation of precise neural tissue structures are not completely understood (Van Essen, 2020).

Neurites are capable of internally generating forces as well as dynamically responding to externally applied forces (Kahn and Baas, 2016). Most classically, extending neurites *internally* generate “pushing” forces via microtubule polymerization near the end of the neurite shaft and via actin polymerization at the lamellipodia and filopodia (Bray, 1979; Goldberg and Burmeister, 1986; Letourneau et al., 1987; Tanaka and Kirschner, 1995; Kandel et al., 2014). In turn, neurites then internally generate “pulling” forces due to actin-myosin activation within the growth cone (Bray, 1979; Letourneau et al., 1987, 1989; Kandel et al., 2014). This mechanism of neurite outgrowth is dependent on traction – generally by specific adhesive ligands – resulting in equal-and-opposite response forces from the substrate and/or through which the growth cone is extending (Lamoureux et al., 1989; Kandel et al., 2014). This complement of internal force-generating machinery underlies the classic “first-phase” of neurite extension and ultimately culminates with location of an appropriate target and subsequent synapse formation (Roos et al., 2000).

Neurites – in particular axons – have been shown to dynamically respond to *externally* applied forces. For example, growth cone mediated towing, as described above to generate long-distance axons, has been mimicked by applying external towing forces (Bray, 1984; Katiyar et al., 2020, 2021; Smith et al., 2022). In one example, “cell puller” machines were shown to extend neurites to a length of up to 960 μm, pulling from the growth cone (Bray, 1984). Postsynaptic axons (i.e., lacking growth cones) also exhibited growth in response to externally applied mechanical tension. This post-synaptic “stretch-growth” of integrated axons was achieved by motion of a towing membrane to move one population of neurons while another population remained stationary, and demonstrated a remarkable 5–10 cm of axonal elongation at the rate of 8 mm/day (Smith et al., 2001, 2022; Pfister et al., 2004; Katiyar et al., 2019). This mechanism of post-synaptic axonal elongation mimics the “second-phase” of axonal growth during development, occurring, for instance, through embryo expansion and ensuing through adolescence due to torso growth and limb lengthening (Smith et al., 2001; Pfister et al., 2004). Subsequently, neurites subjected to slackening have shown length reduction, or relaxation, as quickly as within 60–90 min (Dennerll et al., 1989). Here, axons were able to recover their length after controlled distention by physically moving the growth cone back towards the cell body (Dennerll et al., 1989). Also, this relaxation behavior was observed in integrated (i.e., post-synaptic) axons that were previously subjected to “stretch-growth” after moving the towing membrane back to cause slackening of the axons (Loverde et al., 2011). Together, these examples demonstrate the ability of axons to extend – both passively and actively – in response to externally applied tension, but then also to relax upon subsequent removal of tension. The latter observation suggests the presence of internal mechanisms sensitive to removal of external forces that compensate for reduced internal tension by promoting retraction of axons.

Less appreciated, but no less important, is the phenomenon of axon tract contraction via generation of *internal* forces. In previous studies, researchers have observed axonal retraction *in vitro* in planar culture, most of which were analyzed on a single cell level (Ahmad et al., 2000; Mutalik et al., 2018). However, contraction behavior of fasciculated axonal tracts emerging from a ganglia or an aggregated cell mass has not been studied due to the lack of engineering techniques that provide long-distance three-dimensional (3D) axonal bundles *in vitro*. With the emergence of tissue engineering technology, it is now possible to develop 3D neural tissues using biomaterials and biofabrication techniques to mimic the architecture of native tissue and generate bundled axonal tracts in 3D platforms (Cullen et al., 2012, 2019; Struzyna et al., 2015b, 2017). Over the past several years, our group has developed micro-tissue engineered neural networks (micro-TENNs) using aggregated neuronal populations seeded on the ends of hydrogel micro-columns (Cullen et al., 2012, 2019; Struzyna et al., 2015a,b, 2017; Dhobale et al., 2018; Adewole et al., 2021; Gordián-Vélez et al., 2021a; Burrell et al., 2022). The hydrogel micro-columns in turn supported the growth of long-distance axonal tracts within the lumen to achieve lengths of >2 cm via traditional growth cone mediated axonal extension within a few weeks (Struzyna et al., 2015b; Gordián-Vélez et al., 2021a). The hydrogel micro-column structure provides essential physical support enabling robust, directed axonal growth along with long-term neuronal survival due to the 3D structural architecture. Notably, this general geometry recapitulates aspects of the anatomy of pathways of the brain, specifically the segregated neuronal cell populations spanned by long axon tracts (Cullen et al., 2019).

Modifications in microtubule cytoskeleton and microtubule-associated proteins due to various genetic mutations have been linked to various developmental disorders such as intellectual disabilities, autism spectrum disorders, microcephaly (Barkovich et al., 2012; Hori et al., 2014; Banks et al., 2018; Biswas and Kalil, 2018; Lasser et al., 2018). Most neurological disorders originate during early stages of development, which hinders diagnosis and treatment (Lasser et al., 2018). Complete etiology and pathological conditions of brain developmental disorders and various other neurodegenerative diseases is an evolving area of research, especially human brain disorders due to the lack of appropriate human *in vitro* models. With the advancing tissue engineering techniques, modelling brain pathways under *in vitro* conditions is plausible now to develop testbed for drug screening and understand pathophysiological mechanisms. The approach of generating long-distance axon tracts in 3D has certain advantages over growing the neurons on a 2D surface because the cells behave differently as seen in our previous work (Struzyna et al., 2018; Cullen et al., 2019; Adewole et al., 2021).

Micro-TENNs not only provide a platform to study mechanisms of axonal outgrowth, but also offers a 3D compliant structure to study the physical properties of bundled axonal tracts and their ability to generate and respond to forces. Therefore, in the current study we used micro-TENNs to investigate the presence and mechanisms of axonal tract contraction via intra-axonally generated forces. We also evaluated the ability of these contractile forces to simultaneously provide a source of applied forces to generate axonal “stretch-growth” in adjacent axonal tracts. This study is the first demonstration of internally generated axonal contraction and simultaneous elongation of 3D bundled axonal tracts capable of physically moving dense populations of aggregated neurons with respect to each other. This engineered platform replicates critical features of nervous system development, especially in the gyrencephalic brain where the so-called “axonal tension hypothesis” postulates that axons pull neural tissues as they make connections with different regions and are thus capable of forming gyri when strongly connected areas are drawn together (Van Essen, 1997). As such, this platform may be further exploited to study aspects of axonal cytomechanics, axonal tract maturation, and brain morphogenesis.

## 2 Methods

### 2.1 Fabrication of micro-TENNs

Micro-columns were fabricated using agarose hydrogel molded into a cylindrical structure following the established protocol (Struzyna et al., 2015b; Cullen et al., 2019; Burrell et al., 2022). Briefly, agarose (3% w/v) (Millipore-Sigma, United States, A9539) was dissolved in Dulbecco’s phosphate-buffered saline (DPBS) and the solution was heated at 55°C. Subsequently, the warm liquid solution of agarose was drawn into glass capillary tubes (Drummond Scientific, Broomall, PA) while positioning an acupuncture needle (Seirin, Weymouth, MA) in the center. The agarose solution drawn in the space via capillary action turned to a hydrogel once maintained at RT for 1–2 min. Hollow micro-columns with inner diameter (ID) 160 μm and outer diameter (OD) 345 μm were thus generated by removing the acupuncture needles and gently pushing the agarose micro-columns out of the glass capillary tubes, which were collected in a petri-dish containing DPBS. The micro-columns were then cut to the desired length using forceps. In most of the study, we used 3 mm long micro-columns and in a comparative study we have also used 5–7 mm long micro-columns. Micro-columns were sterilized under UV light for 1 h. An extracellular matrix (ECM) solution was made by mixing rat tail type 1 collagen (Corning, catalogue #354236) and mouse laminin (Corning, catalogue #354232) for a final concentration of 1 mg/mL each. The cocktail collagen-laminin ECM was then pipetted in the lumen of the sterilized hollow micro-columns and set for gelation by placing the ECM-filled micro-columns in a 37°C incubator for 15–20 min. Once the ECM was polymerized into a gel-like structure in the lumen, the micro-columns were ready to add neuronal aggregates to either or both the open ends of the micro-column.

All procedures involving animals were approved by the Institutional Animal Care and Use Committee (IACUC) by the University of Pennsylvania and the Corporal Michael J. Crescenz Veterans Affairs Medical Center and were carried out in accordance with the Public Health Service Policy on Humane Care and Use of Laboratory Animals (2015). Dorsal root ganglia (DRG) were isolated from embryonic day 16 and cortical neurons were isolated from embryonic day 18 Sprague–Dawley rats (Charles River, Wilmington, MA). Briefly, timed-pregnant rats were euthanized using carbon dioxide and the uterus was removed by Caesarian section. Embryos were then retrieved from the amniotic sac and kept in cold Leibovitz-15 medium (Life Technologies, catalogue #11415064). To collect individual DRG, the spinal cords were removed from the embryonic bodies, and DRG were plucked using fine forceps. The DRG thus collected could be directly used to plate on either side of the micro-columns. Similarly, primary cortical neurons were isolated from the brains of the embryos by isolating the cerebrum region of the brains. The cerebral tissue was then dissociated in prewarmed trypsin 0.25% EDTA (Invitrogen, catalogue #25200056) for 12 min at 37°C. After removing the trypsin–EDTA, the tissue was triturated in HBSS (Invitrogen, catalogue #14175079) containing DNase I (Millipore Sigma, catalogue # 11284932001) (0.15 mg/mL). The cells were then centrifuged at 300 g for 5 min and resuspended in neurobasal medium (Invitrogen, catalogue #21103049) + 2% B27 (Invitrogen, catalogue #12587010) + 0.4 mM L-glutamine (Millipore Sigma, catalogue #G7513) and counted prior to making neuronal aggregates. The isolated cortical neurons were then put in PDMS disks with inverted pyramidal wells as described in detail in previous publications to make spherical neuronal aggregates (Struzyna et al., 2015b; Adewole et al., 2021). The well plates containing the neuronal aggregates were incubated for 24–48 h in a mammalian cell incubator (37°C and 5% CO_2_). Prior to placement in the micro-columns, the aggregates were roughly cut using a dissection knife to generate two types of cell mass: a bigger aggregate also referred to as an alpha aggregate and a smaller aggregate referred to as a beta aggregate throughout the manuscript. The cell densities were roughly 10,000–15,000 cells in the alpha aggregate and 5,000 in the beta aggregate.

To fabricate micro-TENNs, the micro-columns prepared previously were placed in an empty petri dish and the ends of the columns were slightly nudged to create space for placing the aggregates. Neuronal aggregates were gently pushed into the end of the micro-column with the help of forceps and dissection knife. Once the aggregate was seeded in the micro-column, media was added to the petri dish, and the cultures were placed in a tissue culture incubator (37°C and 5% CO2) for subsequent days till the termination time-point. The media was changed every 2–3 days by replacing the previous media with fresh, prewarmed media to replenish the culture media. In some instances, the neuronal aggregates were transduced with an adeno-associated virus vector (AAV1.hSynapsin.EGFP.WPRE.bGH, UPenn Vector Core) and (AAV1.CB7.CI. mcherry. WPRE.rBG, UPenn Vector Core) to produce green fluorescent protein (GFP) and mcherry expression in the neurons, respectively. The aggregates were incubated with the respective vectors dissolved in the media (3.2 × 10^10^ Genome copies/mL) prior to seeding in the micro-columns.

### 2.2 Characterization of axon contraction and growth length

The micro-TENNs were imaged at 1, 3–8, 10–13 and 15 days *in vitro* (DIV) using phase contrast microscopy with a Nikon Eclipse Ti-S microscope. The images acquired were analyzed to quantify the neurite growth length and contraction over time using ImageJ/Fiji (Schindelin et al., 2012). Phase contrast images were taken at multiple time-points, and the length of the engineered tissue and the stretch-grown axons were measured. As the axons grew from both directions, integrated axonal bundles were observed as early as 3 DIV; therefore, distance was measured between the two aggregates by measuring the distance between the edges of the opposite aggregates, i.e., the length of the integrated axonal bundle. Change in the length of the axonal bundle was quantified over time to measure the contraction rate, which was measured based on the difference between the length of the axonal bundle at a time point and the previous one relative to the difference in the number of days. We also calculated the length of the stretch-grown axons as the beta aggregate moved farther into the micro-column by measuring the distance between the outermost edge of the beta aggregate and the tip of the longest neurite observed at the edge of the micro-column (where the beta aggregate was originally placed).

Live imaging on GFP-mCherry micro-TENNs was performed at various time-points using a Nikon A1RSI laser scanning confocal microscope and maximum intensity projections (MaxIP) were obtained from the z-stacks. Briefly, live imaging was carried out at room temperature under ambient conditions using short imaging sessions (15–20 min), which did not impact micro-TENN health. Sequential optical sections of 5 μm in the z-plane (40–45 optical sections per ROI) were acquired using NIS elements AR 4.50.00 software and maximum intensity projection (maxIP) images were generated. The integrated axonal bundle length, and stretch-grown axonal length were measured as described earlier based on the live confocal and phase contrast images taken at various time-points. For immunocytochemistry, the micro-TENNs were fixed with 4% paraformaldehyde (PFA) (Electron Microscopy Sciences, catalogue #15710) for 30 min. Prior to staining, the constructs were rinsed with DPBS 4–5 times, and then blocked and permeabilized using DPBS solution containing 4% normal horse serum (NHS) (Thermofisher, catalogue #16050122) and 0.1% Triton X-100 (Sigma, catalogue #T8787). The constructs were then stained with primary antibodies overnight at 4°C. The primary antibodies used in this study were anti-beta-III tubulin (dilution of 1:500; Tuj1; Sigma, catalogue #T8578), MAP2 (dilution of 1:500; Abcam, catalogue #ab254264), and synapsin-1 (dilution of 1:500; Synaptic Systems, catalogue #106011). After washing, the constructs were incubated in Alexa Fluor-conjugated secondary antibody solutions (dilution 1:500; ThermoFisher, donkey anti-mouse 488, donkey anti-rabbit 568 and donkey anti-mouse Alexa-647) for 2–3 h at room temperature. Subsequently, nuclei were counterstained with Hoechst 33342 (ThermoFisher; 1:10,000 in DPBS) for 15 min. The stained micro-TENNs were then imaged using a Nikon A1RSI laser scanning confocal microscope and sequential 5 μm optical sections in the z-plane (40–45 optical sections per ROI) were acquired and maxIP images were generated.

### 2.3 Molecular motor and cytoskeletal inhibitor study

The micro-TENNs were treated with various chemical compounds exhibiting well established, specific inhibition of cytoskeleton proteins and enzymes involved in the motility and growth of neurites. All the inhibitors were dissolved in DMSO first at a defined concentration and then were added in the culture media for 24 h. Phase contrast images were taken before and after 24 h of exposure to quantify the contraction rate to test for effects of the drug on this contraction phenomenon. Rho-kinase inhibitor Y27632 (Cayman Chemical 10,005,583) was added at three different concentrations, namely, 50 μM (*n* = 4), 100 μM (*n* = 3) and 150 μM (*n* = 3). The dynein inhibitor ciliobrevin D (Sigma 250,401) and the microtubule-destabilizing agent nocodazole (Sigma M1404) were added at 150 μM (*n* = 3) and 20 μM (*n* = 4) respectively. The untreated micro-TENNs acted as a control group (*n* = 4), whereas a 0.6% DMSO group (*n* = 3) was included to control for the effects of the vehicle. Between 4–7 DIV, a time interval in which the aggregates have already connected and we could ensure contraction was happening, drugs were administered to determine their effect on contraction by measuring the length change after a 24 h treatment period.

### 2.4 Statistical analysis

All statistical analyses were performed using GraphPad Prism 10 software (GraphPad Software, Inc). Data are graphically represented as mean +/− standard error of the mean (SEM) as indicated in the figure captions. For the drug treatment test, data was transformed using the formula [1/(Y + 1)] to achieve normality of the data before applying one-way ANOVA and significance was evaluated using 0.05 as the cutoff *p*-value.

## 3 Results

### 3.1 Development of micro-tissue engineered neural networks (micro-TENNs)

**Figure 1:**
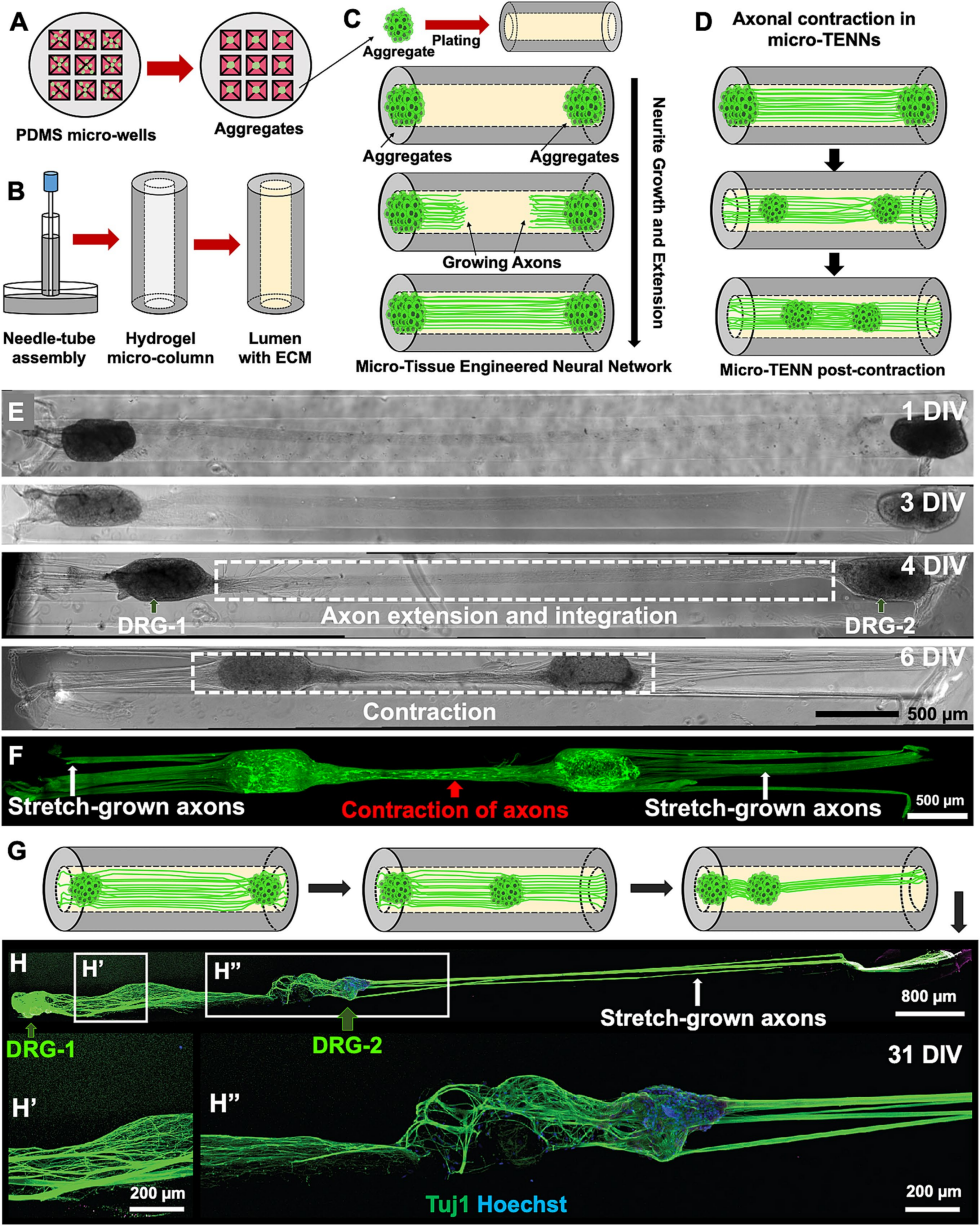
Overview of the micro-tissue engineered neural networks (micro-TENNs). **(A)** Schematic representation depicts the fabrication process of micro-TENNs using neuronal aggregates generated in customized 3D micro-wells inspired by the natural dorsal root ganglia (DRG). **(B)** The needle-capillary tube assembly is represented to illustrate the fabrication process of hollow agarose micro-columns (outer diameter: 345 μm, inner diameter: 160 μm), which is later filled with an extracellular matrix (ECM) composed of collagen type 1 (1 mg/mL) and laminin (1 mg/mL). **(C)** The micro-TENNs were fabricated by seeding the aggregated neurons in both the ends of the micro-column to allow extension of long-distance axonal projections throughout the lumen. **(D)** Illustration of the contraction of integrated bundled axons observed in a micro-TENN. **(E,F)** Example of a homogenously contracted micro-TENN developed using DRG plated on both the ends of a micro-column showing the growth stages from 1–6 DIV; the axons contracted (red arrow) between DRG-1 and DRG-2 while simultaneously applying internal forces to “stretch-grow” axons from the other side (white arrows). The micro-TENN is labeled using Calcein AM to denote live cells and axons. **(G,H)** Example of heterogeneously contracted micro-TENN showing displacement of one aggregate (DRG-2; right) in the inner lumen towards the left showing elongated stretch-grown axons (white arrows) in the micro-column. The micro-TENN is stained with anti-Tuj1 (green) and Hoechst nuclear marker (blue) through the process of immunocytochemistry (ICC). Scale bars: **E,F**: 500 μm, **H**: 800 μm, **H′** and **H″**: 200 μm.

We previously developed micro-TENNs as miniature hydrogel-based neuronal constructs that provide a platform to generate long-distance aligned axonal bundles from segregated neuronal populations within a constrained tube-like structure (Cullen et al., 2012; Struzyna et al., 2015a,b, 2017; Adewole et al., 2021). With neuronal somata residing only at one end of the micro-column, the axonal tracts could be grown in a single direction according to the length of the column as shown previously (Adewole et al., 2021). These unidirectional long axon bundles were intended to recapitulate aspects of the structure of native neuronal-axonal pathways in the brain, which may enable these constructs to replace lost brain pathways after implantation (Struzyna et al., 2015b, 2018, 2022; Adewole et al., 2021). To further study micro-TENNs as *in vitro* testbeds, we developed bidirectional micro-TENNs with neuronal aggregates seeded at both ends, and neurites extending within the interior lumen of the micro-column within 3–5 days *in vitro* (DIV) as represented in the schematic diagram (Figures 1A–D).

Initial studies were carried out using DRG as the cell mass to examine the behavior of axonal growth in a bidirectional construct. Since DRG vary in size, we observed variation in the mobility of the DRG aggregates during and after axonal extension within the lumen of the micro-column. After axonal integration between both DRG was achieved, we observed contraction of the axonal tracts spanning the DRG, and, depending on the size of the DRG relative to the lumen diameter, this phenomenon was sufficient to pull both DRG further into the lumen of the hydrogel and towards each other (schematic representation in Figure 1D; representative images in Figures 1E,F). In addition to the contraction of the interior axonal bundle, we also observed stretch-grown axons from the opposite (exterior) sides of the DRG, thereby showing growth response of axons based on displacement of their cell bodies. In other instances, only one DRG was pulled towards the center of the micro-column, whereas the other DRG remained stabilized at the end of the column, indicating simultaneous axonal contraction from the internal side, while axons extending from the external side of the displaced DRG were stretch-grown (Figures 1G,H). Although we observed long-distance stretch-grown axons on the external side of the displaced DRG, the axons between the two DRG seemed relaxed in places (Figure 1H″) and not tightly contracted as seen in Figure 1F or Figure 1H′. This could be attributed to a mechanical disturbance while handling the microtissue. Overall, this initial DRG micro-TENN study served as a foundation to investigate the contraction phenomena in 3D bundled cortical axons.

### 3.2 Development of cortical neuron bidirectional micro-TENNs

**Figure 2:**
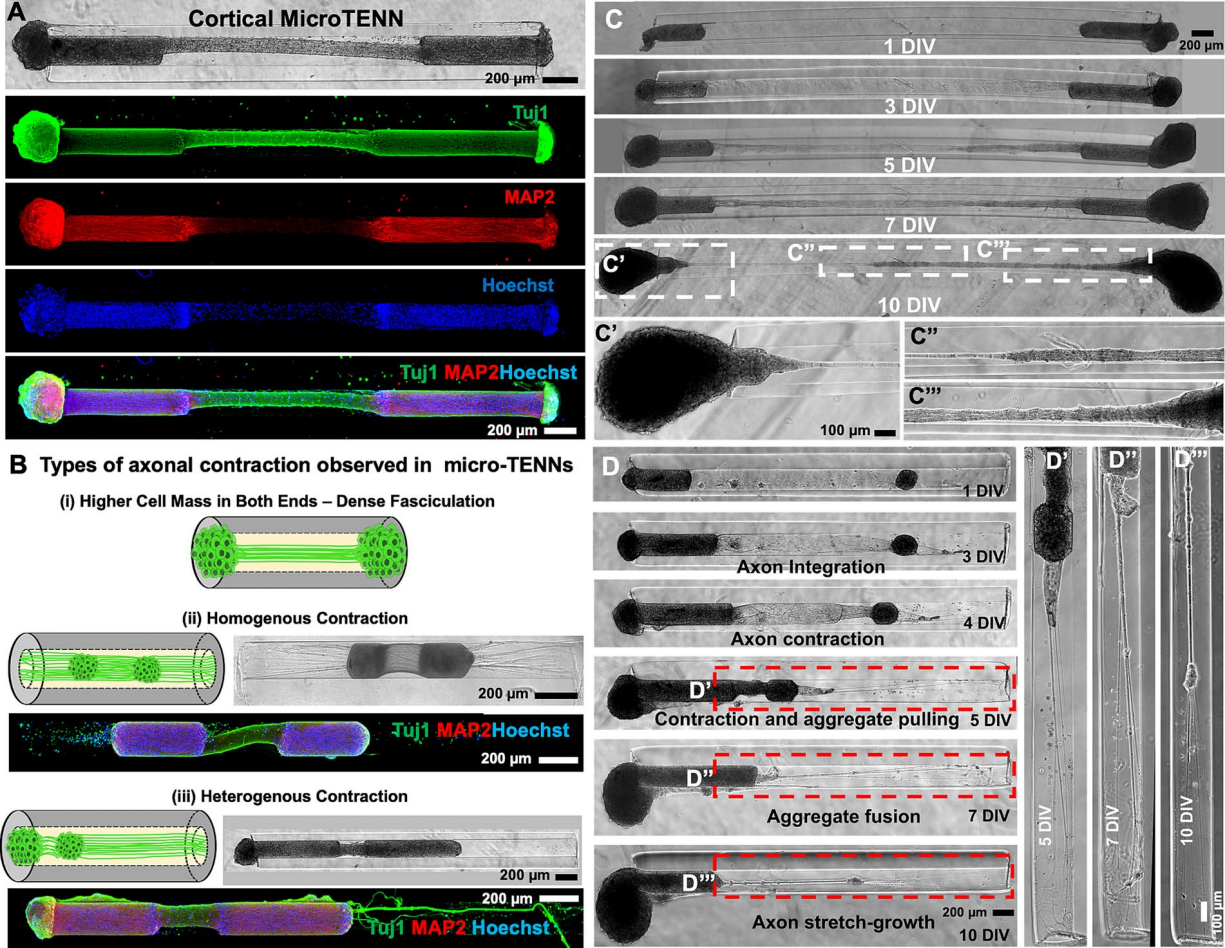
Cortical micro-TENNs as a biofidelic testbed. **(A)** Cortical micro-TENNs were developed by aggregating primary rat cerebral cortical neurons as represented by the phase contrast image. The ICC images represent the characteristic markers of cortical neurons stained using anti-Tuj1 (green), anti-MAP2 (red) and Hoechst nuclear marker (blue). **(B)** Optimization done using aggregate size to develop cortical neuronal biofidelic testbed shows different axo-somatic distribution in various constructs: (i) Dense fasciculation (ii) homogenous contraction, and (iii) heterogenous contraction. Representative ICC images illustrate MAP2 (soma, red), Tuj-1 (all neurites, green) and Hoechst staining (nuclei, blue). **(C)** Representative phase contrast micrographs of the micro-TENNs developed by seeding equally sized larger aggregates at both the ends showing dense axonal fasciculation at 10 DIV. **(D)** Representative phase contrast images of the construct at various time-points show heterogenous contraction in the micro-TENN developed by seeding unequal aggregates on both sides: larger and smaller aggregates referred to as alpha (left) and beta (right), respectively. **(D′–D″′)** Magnified regions of the micro-TENN represent subsequent axonal stretch growth at various time-points. Scale bars: **A–D**: 200 μm, **C′–C″′**, **D′–D″′**: 100 μm.

After developing the bidirectional micro-TENN platform and observing the contraction phenomenon in DRG, we sought to study axonal contraction in primary cerebral cortical neurons when grown as bidirectional micro-TENNs. Figure 2A represents the typical structure of a cortical micro-TENN labeled with MAP-2 and beta-tubulin III (Tuj-1). To emulate the ganglia structure of DRG, we made cortical neuronal aggregates from dissociated primary cerebral cortical neurons and seeded them at both ends of the micro-column (Struzyna et al., 2017). As seen in the phase contrast image of a cortical micro-TENN (Figure 2A), the two aggregates on each side are connected and spanned by bundled axonal tracts. The ICC images show an axonal bundle expressing Tuj-1, whereas the aggregates (on both ends) show MAP2 and Hoechst+ nuclei along with Tuj-1 (Figures 2A,B). The cortical micro-TENNs demonstrated axonal growth within the lumen of the column between 3–7 DIV (Figures 2C,D). As the cortical axon tracts grew over time and achieved putative synaptic integration, we could observe axonal contraction of the bundled axon tracts (Figures 2C,D).

Based on the observation that axonal contraction caused displacement of individual DRG within the lumen (Figures 1F,H), we next explored the effect of the cortical neuronal aggregate size on their contraction behavior in micro-columns (Figure 2B). On plating equally sized aggregates, we sometimes observed fasciculation of the axonal bundles as shown by thinning of the bundles by 10 DIV but no contraction like that observed in DRG micro-TENNs (comparison between Figures 1E, 2C). Here, the growing size of both the aggregates may have contributed to this effect, as the bulk of the aggregate remained on the exterior of the micro-column post axonal integration. However, dense fasciculation was visible in the zoom-in images at 10 DIV showing axonal tension between the two aggregates (Figures 2C′,C″). Together, we observed three types of axonal fasciculation/contraction as schematically represented (Figure 2B): (1) large aggregates at both sides – the aggregates remained at the ends of the micro-column but could not be pulled inwards due to the aggregate-size (Figure 2C′); (2) small aggregates at both sides – both aggregates were pulled inwards homogeneously spanning similar lengths; and (3) heterogeneously sized cell masses at either side – one bigger (alpha) aggregate and one smaller (beta) aggregate seeded on the opposite ends of a micro-column leading to heterogenous contraction of the axons. This last group is represented in Figure 2D showing the growth of neurites at various time-points. As seen in the phase contrast images, the axons from alpha (left) and beta (right) integrated with each other at 3 DIV and the axonal tract began contracting at 4 DIV, which pulled the beta aggregate to the interior, before both the aggregates fused together at 10 DIV (Figure 2D). The displacement of the beta aggregate from one end of the column towards the alpha aggregate led to stretch-growth of axons extending from the outermost side of the beta aggregate and their anchor point at the end of the column (Figure 2D′–D″′). Understanding the relationship between aggregate size (relative to lumen diameter) and subsequent displacement aided in selecting experimental conditions to further study axonal contraction and subsequent elongation of primary rat cortical neurons within the micro-columns.

### 3.3 Characterization of axonal contraction and stretch-growth in cortical micro-TENNs

**Figure 3:**
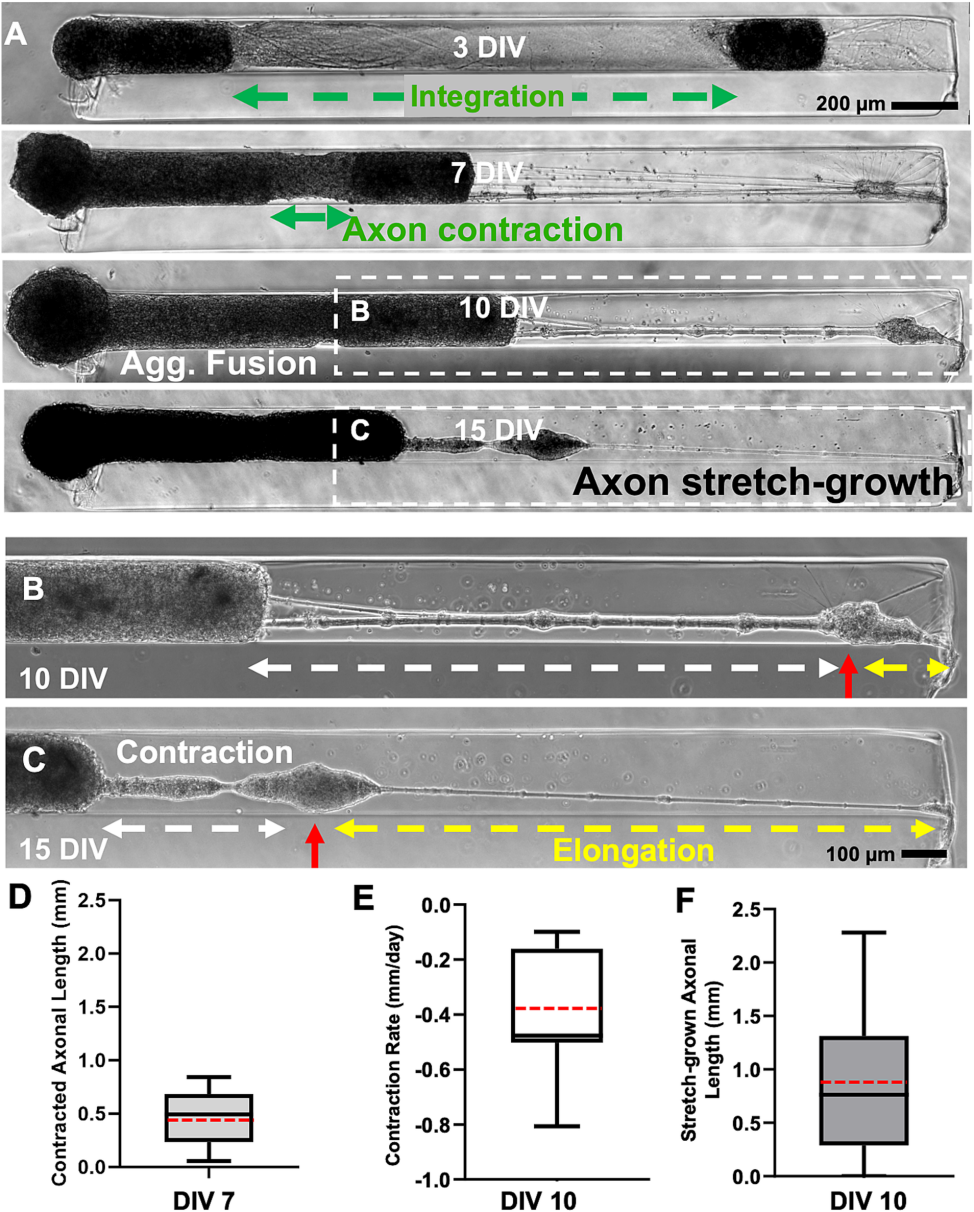
Characterization of heterogenous contraction in cortical micro-TENNs testbed. **(A)** Representative phase contrast microscopic images of a full-length 3 mm long construct demonstrate heterogenous contraction at 7 DIV and elongated stretch-grown axons at 15 DIV in the testbed developed by seeding aggregates of different size at both the ends of the micro-column (alpha aggregate on the left and beta aggregate on the right end). **(B,C)** The magnified images show secondary contraction in the stretch-grown axons (highlighted by red arrow) between 10 and 15 DIV further represent the motility in the axonal bundle. Quantification of the contraction and elongation of the axons was carried out between 7 and 10 DIV prior to the fusion of aggregates and the data was plotted showing a range using box and whisker plots. **(D)** The length of the contracted integrated axonal bundle between the two aggregates within each construct measured at 7 DIV (*n* = 15). **(E)** The contraction rate at 10 DIV relative to 7 DIV for the contracted bundled axons between the two aggregates (*n* = 15). **(F)** The length of the stretch-grown axons measured at 10 DIV for various constructs (*n* = 15). The box plots represent median (black horizontal line inside the box), average mean values (red dashed lines), interquartile range (box) between the first and the third quartiles, and whiskers (error bars = min/max values). Scale bars: **A**: 200 μm, **B,C**: 100 μm.

To better understand the contraction and elongation of cortical axonal bundles within the constrained tubular dimensions, we seeded neuronal aggregates of different sizes (alpha/beta) at the opposite ends of a 3 mm long micro-column to generate a heterogenous bidirectional cortical micro-TENN. In addition, the alpha aggregate was not seeded completely inside the lumen to keep it immobilized at the edge of the micro-column as it grew, whereas the beta aggregate was seeded completely inside the column (Figure 3). The phase contrast images taken over time display the beginning of axonal integration from one side and stretch-growth from the other side of the beta aggregate as shown in the representative images (Figures 3A–C).

The contraction and stretch-growth of the axons was attributed to the innate tension between the integrated axonal bundle and the aggregates given that there was no external force being applied. In many instances, the contraction of the tissue was sufficient to cause the complete fusion of both aggregates (10 DIV in Figure 3). The length of the integrated axonal bundles decreased over time, showing an average length of only 0.46 ± 0.26 mm at 7 DIV (Figure 3D). The average contraction rate was found to be-0.38 ± 0.21 mm/day when calculated at 10 DIV with respect to 7 DIV (Figure 3E). The contraction resulted in more than 50% reduction of the initial axonal length as indicated by the 7 DIV phase image. The length of the stretch-grown axons varied depending on the time of maximum extension of axons post-fusion, which was mostly observed at 10 DIV. Hence, we calculated the length of the stretch-grown axons at 10 DIV and found their lengths varied in a wide range between 0.29 mm to 2.28 mm in various constructs (*n* = 15) (Figure 3F). This wide distribution of axonal lengths after stretch-growth at a particular time-point was attributed to the fact that the time-point of onset of contraction and of stretch-growth varied from construct to construct. In some instances, contraction was such that the stretch-grown axons were also pulled in along with the fused aggregates (Supplementary Figures S1, S2).

**Figure 4:**
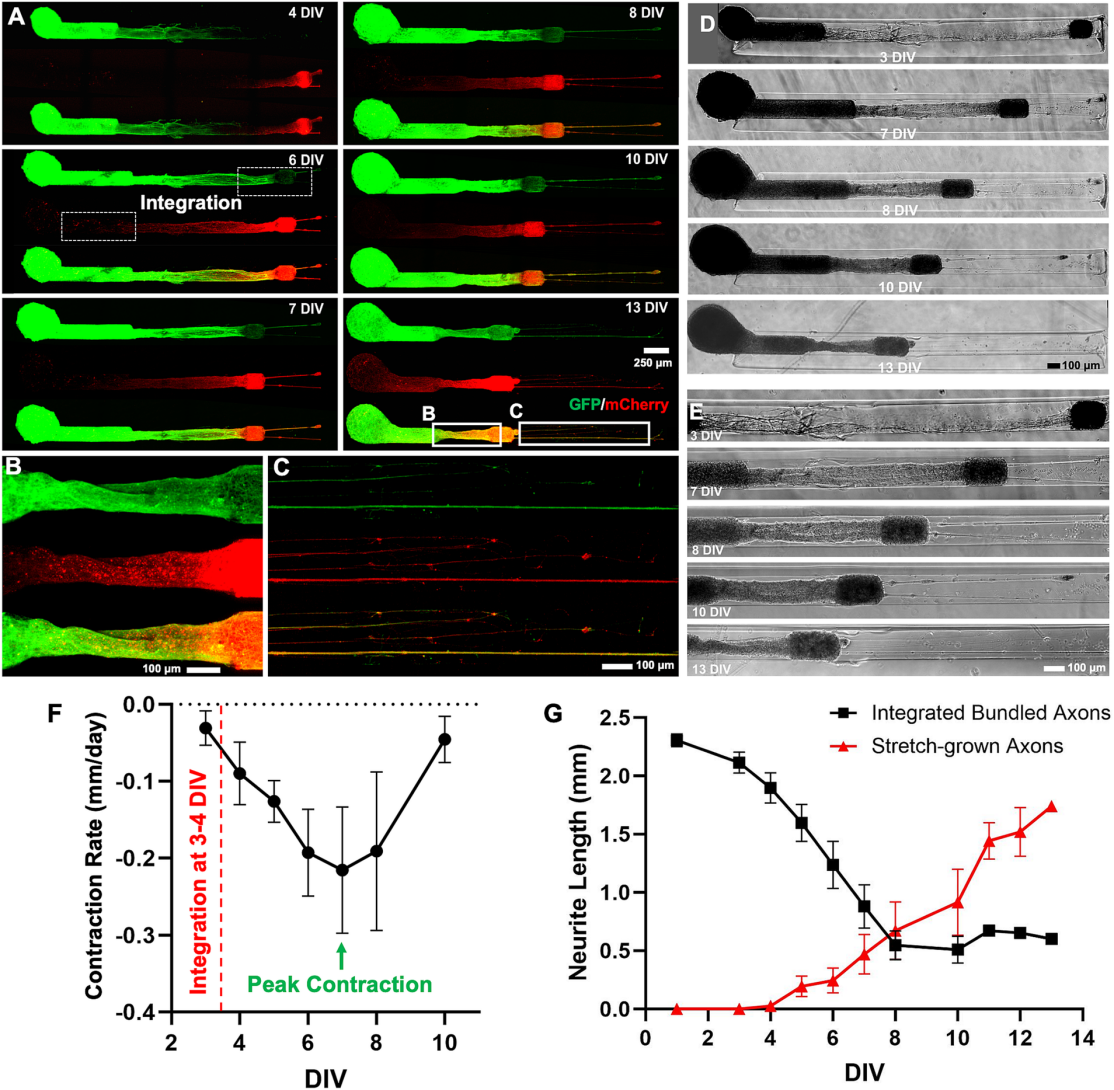
Characterization of heterogenous contraction in GFP-mCherry labelled micro-TENNs. **(A)** Confocal 3D reconstructions of a micro-TENN with aggregates expressing AAV transduced GFP or mCherry vectors. Live images of the growing construct illustrate the same regions over time to show the neuronal changes occurring during 4–13 DIV. The micro-TENN with labelled alpha aggregate (GFP; left) and beta aggregate (mCherry; right) demonstrates the integration of axonal tracts at 6 DIV. **(B,C)** The inset images display zoom-in regions of the integrated contracted axonal bundle between the two aggregates and stretch-grown axons post-contraction at 13 DIV. **(D)** Phase contrast images of the same construct showing growth over time from 3 to 13 DIV. **(E)** Zoom-in phase images of the same construct illustrates the changes in the axonal regions as the integrated bundle contracted and stretch-grown axons were elongated. **(F)** The contraction of the bundled axons measured over time allow calculation of the rate of contraction, showing that the peak contraction rate occurred at 7 DIV under these fabrication parameters (*n* = 5). **(G)** The line plot demonstrates change in the length of integrated axonal bundle and stretch-grown axons at various time-points from 1 to 13 DIV (*n* = 5). Data are represented as mean ± SEM. Scale bars: **A**: 250 μm, **B–E**: 100 μm.

To further characterize the mechanism of the contraction, the alpha and beta aggregates were transduced with AAV vectors to express GFP and mCherry, respectively. An example of a heterogenous micro-TENN (3 mm long) with a GFP+ alpha aggregate and a mCherry+ beta aggregate is shown in the confocal images taken at 6 DIV when the axonal integration was significantly visible (Figure 4A). Images taken at 6 DIV depict stable integration between aggregates and the beginning of the contraction of the axonal bundle as exhibited by the slight displacement of the beta aggregate (red) towards the alpha aggregate (green) along with generation of stretch-grown axons. Live imaging done at various time-points also confirmed that the bundled axons consisted of a mixture of axons coming from both sides and that the length of the integrated bundle reduced over time. The zoom-in image shown in the inset image at 13 DIV (Figure 4B) illustrates integration in the aggregates as seen by the presence of GFP+ axons integrating with the mCherry+ cell mass and vice versa. An overlap of green and red channels was also observed on the right side of the beta aggregate depicted in inset image (Figure 4C) suggesting that the GFP+ axons originating from the alpha aggregate were able to grow further after crossing the beta aggregate region. In other instances, we found axons stretching from only the beta aggregate (no overlapping of green and red channels in Supplementary Figure S3).

**Figure 5:**
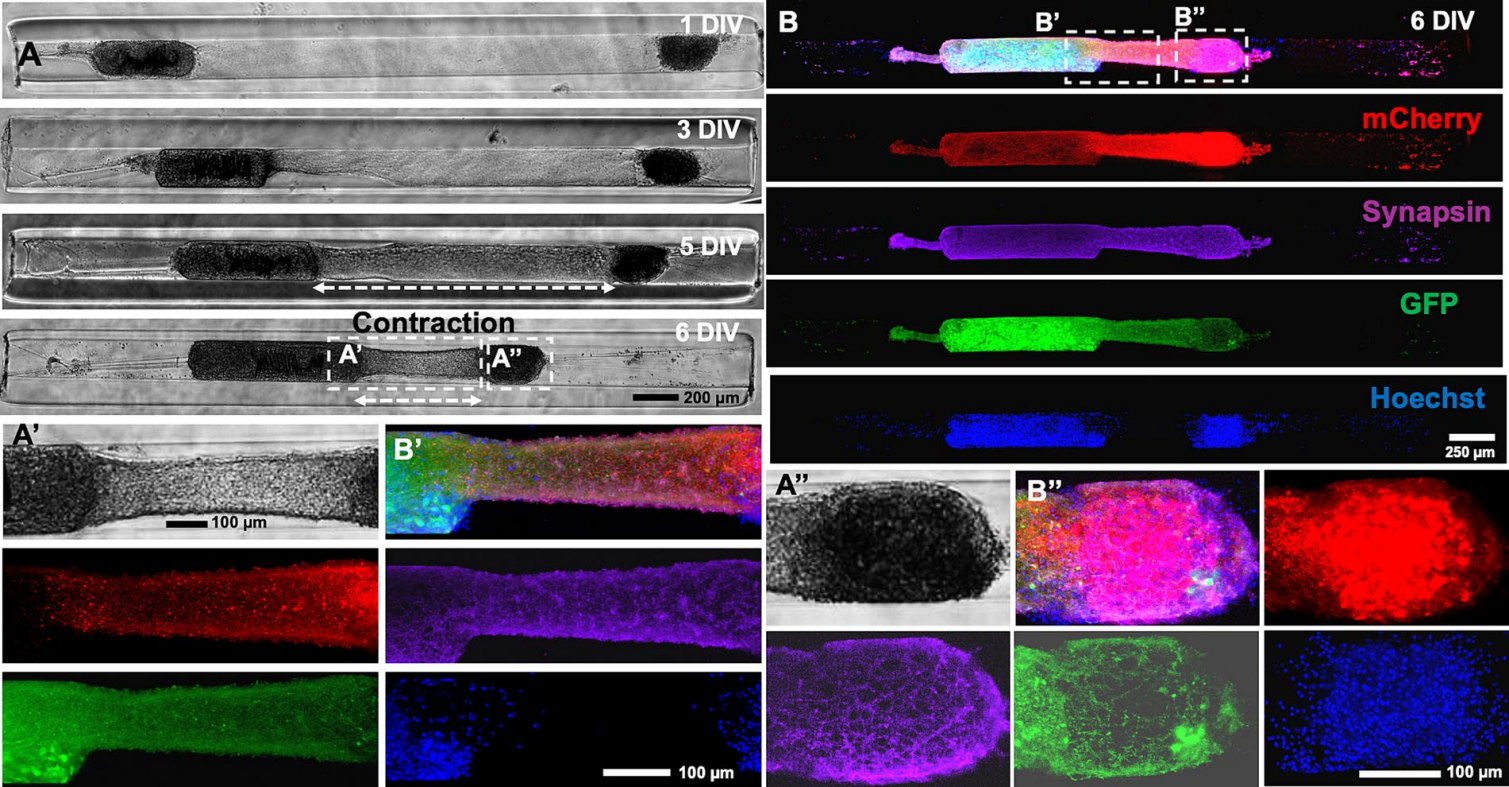
Putative synaptic integration in heterogenous cortical micro-TENNs. Micro-TENN cytoarchitecture was observed by immunolabelling the micro-TENNs at 6 DIV. **(A)** Representative phase contrast images of the construct at various time-points. **(B)** Confocal reconstruction of cortical micro-TENNs after immunocytochemistry (ICC). Cells in the micro-TENN transduced with AAV vectors expressed GFP and mCherry, which were further stained for presynaptic marker – synapsin-1 (far red) and nuclei (Hoechst; blue) to demonstrate the presence of pre-synaptic terminal protein Synapsin-I in the neural microtissue as the axons integrated and contracted. Zoom-in phase contrast images **(A′,A″)**. The stained micro-TENN with zoom-in images show a labelled bundled axonal region **(B′)** and cell-aggregate region **(B″)** including individual channel images. Scale bars: **A**: 200 μm, **B**: 250 μm, **A′,A″, B′,B″**: 100 μm.

On quantifying the contraction rate using the confocal images taken at various time-points, the peak of the contraction occurred at 7 DIV (Figure 4F). Herein, we further measured the length changes happening from 1 to 13 DIV, which showed that the length of the integrated bundled axons (axonal length between the two aggregates) reduced from 2.33 mm at 1 DIV to a minimum length 0.60 mm at 13 DIV, whereas the length of stretch-grown axons increased from 0 mm at 1 DIV to maximum 1.7 mm at 13 DIV within the 3 mm long construct (Figure 4G). Next, we performed immunocytochemistry on the contracted micro-TENNs at two time-points, namely, 5 DIV (time point of axonal integration) and 6 DIV (time point of axonal contraction) to confirm axonal integration and putative synaptic connections between the aggregates. The micro-TENNs demonstrated the presence of synapsin-1 throughout the length of the micro-TENN at 6 DIV (Figure 5). The zoom-in images show the axons from alpha aggregate (GFP+; green) integrating with the beta aggregate (mCherry+; red) along with extensive expression of the presynaptic terminal protein synapsin-I (Figures 5B′,B″). The 5 DIV ICC images depicted the same with robust colocalization of synapsin-I with GFP neurites projected over the mCherry aggregate at the time of axonal integration (Supplementary Figure S3), implying presence of putative synaptic connections within the bundled axons.

In order to further investigate the contraction pattern of the integrated axons with respect to the length of the micro-column, we developed 5–7 mm long micro-TENNs and compared the observed contraction with that of the 3 mm long micro-TENNs detailed above (Supplementary Figure S5). Here, we selected homogenous micro-TENNs, i.e., aggregates of equal size were seeded at both the ends of the micro-columns. The aggregates were seeded to be completely inside the lumen, so they were not immobilized at the edges of the column. The 3 mm long micro-TENNs demonstrated axonal integration at 3 DIV and began phases of contraction early on with complete fusion of both the aggregates by 10 DIV (Supplementary Figure S5A). In comparison, the same events were delayed for 7 mm long micro-TENNs; axonal integration and contraction were first observed around 8 DIV (Supplementary Figure S5B). Since the micro-TENNs were only cultured for 21 days, we did not observe aggregate fusion by 21 DIV, but noticeable axonal contraction was seen between 10 DIV and 21 DIV. ICC images corroborated the presence of contracted axons between both aggregates in the 3 mm micro-TENN at 10 DIV (Supplementary Figure S5C). In comparison, the 7 mm long micro-TENN showed presence of a predominant Tuj-1+ axonal bundle between the two aggregates at 21 DIV (Supplementary Figure S5D). Of note, the presence of MAP2+ neurites between the aggregates in the 7 mm long micro-TENN could be a result of axons being less mature as there may be a delay in the post-synaptic maturation process (Supplementary Figure S5D).

### 3.4 Mechanisms of contraction in cortical micro-TENNs

**Figure 6:**
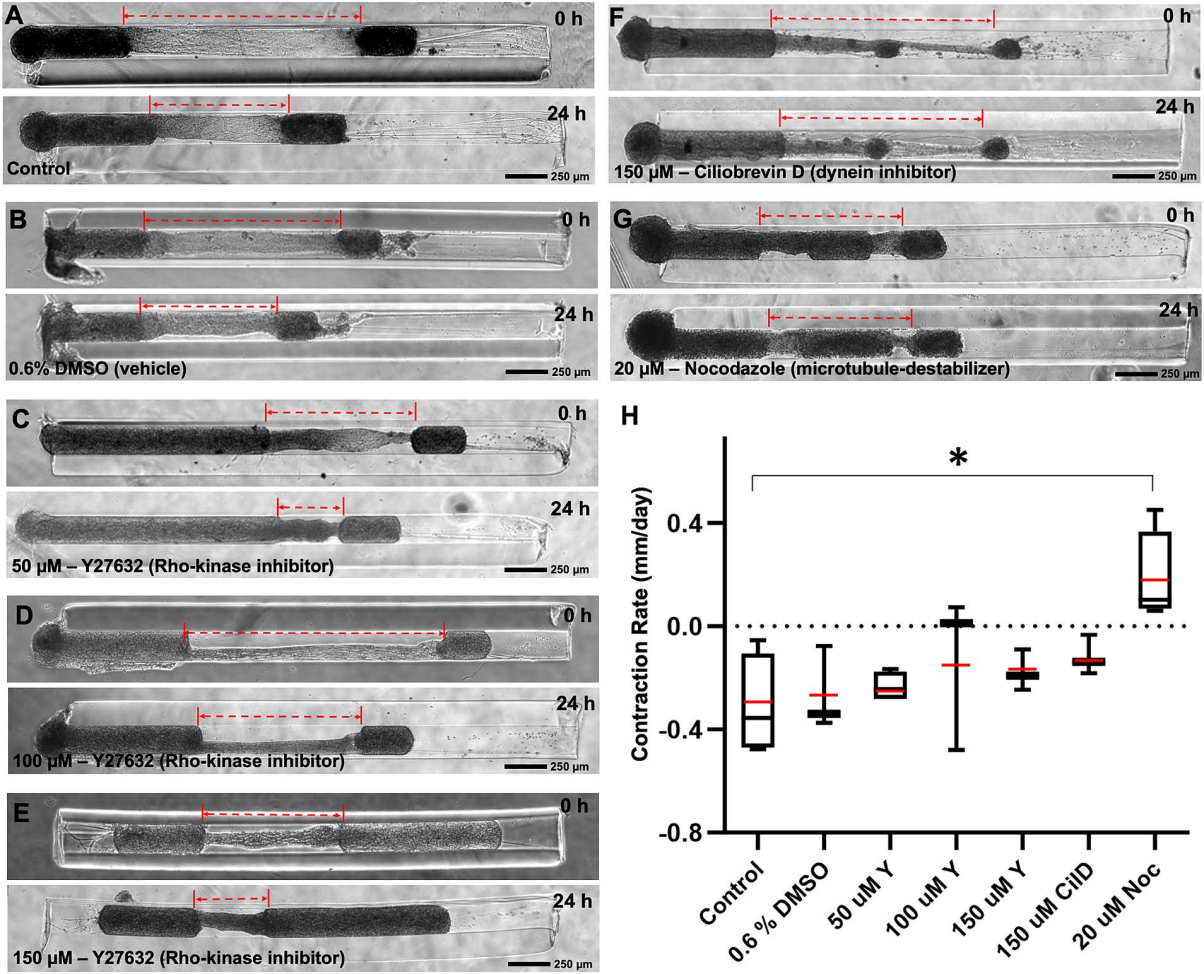
Mechanism of contraction in heterogenous cortical micro-TENNs. Drug effect on the contraction behavior of axon bundles in micro-TENNs. Phase contrast images of the micro-TENNs are shown before and 24 h post-treatment for various groups (*n* = 3 or 4): **(A)** Control (only media; *n* = 4), **(B)** Vehicle control with 0.6% DMSO in the media (*n* = 3), **(C–E)** Y27632 Rho-kinase inhibitor treatment at 50 μM (*n* = 4), 100 μM (*n* = 3), and 150 μM (*n* = 3) concentrations, **(F)** Ciliobrevin-D dynein inhibitor treatment at 150 μM concentration (*n* = 3), and **(G)** Nocodazole microtubule inhibitor at 20 μM concentration (*n* = 4). The dotted red lines highlight the length of the integrated axonal bundle between the two aggregates before and after drug administration. **(H)** The contraction rates from the various treatment groups were calculated after the 24 h treatment relative to the previous time-point, and are represented using a box and whisker plot. The study demonstrates various effects of the treatments in attenuating the rate of axonal contraction, showing significant suspension by nocodazole (*p* < 0.05) in comparison to the control. The box plot represents median (black horizontal line inside the box), mean (red lines), interquartile range (box) between first and third quartiles, and whiskers (error bars = min/max values). Statistical analysis was performed by transforming the data with formula [1/(Y + 1)] as represented by Supplementary Figure S6 and applying one-way ANOVA. Scale bars: **A–G**: 250 μm.

To investigate the contribution of various well-known molecular motor proteins to the mechanism(s) driving contraction, the contracting micro-TENNs were treated with different pharmacological inhibitors for 24 h. We employed Y27632, Ciliobrevin-D (Cil-D), and Nocodazole, which are a Rho-kinase inhibitor, a dynein inhibitor, and a microtubule destabilizer, respectively. The change in the length of micro-TENNs was calculated before and 24 h after exposure to these drugs at various concentrations (Figure 6). We found that the contraction rate after 24 h was unaffected by the exposure to DMSO (the vehicle used for all the compounds). The rho-kinase inhibitor Y27632 was evaluated at three different concentrations, namely, 50, 100 and 150 μM. However, we did not see any dose response or significant differences in comparison to the control groups. Interestingly, the dynein inhibitor Cil-D (150 μM) produced a trend towards attenuating axonal contraction. Here, the mean contraction rate was found to be-0.12 mm/day in the Cil-D treatment in comparison to-0.31 mm/day in the control group, but this change was not statistically significant. However, administration of the microtubule destabilizing drug nocodazole (20 μM) produced a significant change in the mean contraction rate, which this agent effectively reversed to a level of 0.18 mm/day. This showed that contraction was primarily dependent on microtubule stability of the axons.

## 4 Discussion

Forces deriving from within axons via *internal* mechanisms as well as these being applied to axons from *external* factors have a profound influence on the structure of the CNS. Indeed, axonal extension and axonal pruning continuously affect network formation and brain morphogenesis during nervous system development (George et al., 1988; Heidemann et al., 1995; Silbereis et al., 2016). The regulation of resting tension in cortical convolutions allows the large volume of neural tissue to fit in the much smaller cranial space (George et al., 1988; Heidemann et al., 1995; Silbereis et al., 2016). As the evidence indicates, genetic mutations leading to disruptions in mechanical cues and/or mechanosensation have been linked to neurodevelopmental diseases like autism, attention-deficit hyperactivity disorder, learning disabilities, cerebral palsy and impairments in vision or hearing (Silbereis et al., 2016; Ayad et al., 2019). This has led to a critical need for developing biofidelic tissue models *in vitro* to improve our understanding of brain functions and identify factors attributing to such pathological conditions. As such, a 3D tissue model holds immense potential in recapitulating the complex structural and functional properties of large-scale neural circuitry. Over the past decade, our lab has explored several neural tissue engineering approaches to model various brain pathways and structures using primary rat neurons (Struzyna et al., 2015a,b, 2017, 2018, 2022; Harris et al., 2016; Adewole et al., 2021) and/or human neurons (Cullen et al., 2019; Gordián-Vélez et al., 2021a). We have also investigated the morphological and cytoskeletal reorganization that occurs in the bundling of astrocytes (Winter et al., 2016; Katiyar et al., 2018; O’Donnell et al., 2018, 2021; Purvis et al., 2022) or Schwann cells (Panzer et al., 2020) after seeding in micro-columns.

In order to study aspects of mechanical tension in bundled axonal tracts *in vitro*, we leveraged our previously developed micro-TENN platform that was fabricated using aggregated primary rat cortical neurons within custom-built hydrogel micro-columns. In these bidirectional micro-TENNs, neuronal aggregates were seeded on both ends of the micro-columns and subsequently extended axonal bundles from both sides that eventually physically integrated the two neuronal populations. We observed that once the axons crossed over and connected, the bundled axons could contract significantly, and this pulled the aggregates long distances toward each other within the lumen of the micro-column (as demonstrated in Figures 2–5). The same phenomenon was also observed when micro-TENNs were developed using DRG in the initial study (see Figure 1). The design of the micro-TENN provides two important structural features in developing a testbed to study axonal biomechanics: (1) hollow agarose outer shell as a structurally stable encasement lacking direct cell adhesion moieties, and (2) a central soft collagen-laminin ECM filling providing mobility and matrix remodeling capability to the growing neurites. Together, the micro-column testbed provided an optimum 3D structural platform to hold the cell body mass at one end while offering tractability to dynamic axonal tracts within the lumen. Also, the compliant nature of collagen-laminin ECM in the lumen likely provides flexibility to the growing/contracting neurites as the ECM would not remain adhered to the walls of the agarose hydrogel. The ECM biomaterial of the lumen plays a significant role in the growth rate of axons as observed in our previous study when the collagen + laminin ECM cocktail was compared with collagen alone or chemically crosslinked collagen (Struzyna et al., 2018). Therefore, the axonal contraction rates achieved in this system would likely be influenced by the ECM constituents, and it would be worthwhile to characterize these effects in future studies. Similarly, changing the composition of the hydrogel micro-column would also influence the axonal contraction rate as the stiffness provided by the outer shell varies depending on the biomaterial (Gordián-Vélez et al., 2021b). We have previously seen an effect on the growth rate of primary rat dopaminergic neurons by changing the micro-column from agarose to hyaluronic acid (Gordián-Vélez et al., 2021b). Cell-material interactions could further play a role in tissue mechanics if the hydrogel micro-column provide adhesion sites to the soma when the neural network is formed. Consequently, selecting a single platform using agarose hydrogel as the outer shell and collagen-laminin cocktail as the ECM, we carried out the present study by just manipulating the size of cortical neuronal aggregates on either side of the construct to better control and subsequently analyze the contractile behavior of the bundled axon tracts.

Spontaneous axonal contraction has been identified as a mechanism for maintaining resting tension in chick sensory neurons cultured on 2D glass slides (Mutalik et al., 2018). The authors hypothesized that adhesive sites initiated a mechanical feedback response to enhance axonal contractility. We observed a similar phenomenon in the micro-TENNs developed using DRG as the axons grew and, upon integration (i.e., finding a target population), contracted significantly to maintain resting tension. The correlation can be seen between the contraction behavior observed in previous studies using chick sensory neurons and the contraction of bundled sensory axons in DRG micro-TENNs in our pilot study (Figure 1). This might also demonstrate relevance to aspects of PNS development as this behavior is essential in various neurodevelopmental processes, which include stretch-growth of axons as well as length minimization or contraction of certain neural networks during the growth of an organism.

Indeed, as axons grow and reach a certain length, they develop a resting tension that is often regulated by the motor-generated intrinsic forces affecting mechanotransduction in the cells (Mutalik et al., 2018). Of note, we observed cell migration along integrated axonal tracts in our system, ultimately filling the luminal space between the aggregates as seen in the phase contrast images at later time points (e.g., see Figure 3A). This cell migration could also be contributing to an overall resting tension of the overall 3D construct at later time points. However, this cell migration was secondary to axon contraction as the axonal bundles contracted as early as 3 DIV in some instances once the axons integrated (Supplementary Figure S2). Nonetheless, this system may be used in future studies to examine the mechanisms and biophysical consequences of neural cell migration along 3D axonal tracts.

Inherent contractile mechanisms can also be seen in the behavior of neurons with severed axons, which shows that axotomy often leads to retraction of the remaining length of axons demonstrating a primary role of the soma in maintaining a resting tension (Shao et al., 2019). The study revealed that there is an interplay between neuron-substrate adhesion and the pre-tension in the axons, thereby impacting the overall retracted length of severed cortical axons (Shao et al., 2019). Retraction rate has also been found to be dependent on the distance of transection from the perikaryon in a study showing lower survival rate of neurons when transected near the cell body (Cengiz et al., 2012).The retraction rate of DRG axons ranged from 0.35 ± 0.02 mm/day to 1.52 ± 0.29 mm/day depending on the distance of transection from the cell body (Cengiz et al., 2012). This reported range is similar to the mean axonal contraction rate of 0.38 mm/day found in our system at 10 DIV (Figure 3E); however, note that the previous report was for severed axons and the contraction mechanism could be different than in our system that features intact axons.

To reliably reproduce the contraction phenomenon, it was critical that the fabrication of the cortical neuronal network be modified to allow heterogenous contraction and axonal extension from both the ends by using neuronal aggregates of different sizes (Figure 2B). The micro-TENNs demonstrated integration of the axonal bundle at 3 DIV as the axons from both sides crossed over (Figure 3; Supplementary Figure S2), displaying sufficient growth of cortical axons within 3 days. A detailed study previously published by our group reported a mean axonal growth rate of 0.56 ± 0.03 mm/day in bidirectional cortical neuron micro-TENNs in comparison to 0.039 ± 0.02 mm/day for cortical neurons in 2D planar cultures at 3 DIV (Adewole et al., 2021). That study also showed a minimum growth rate of cortical axons as 0.32 ± 0.04 mm/day in the 3-mm long micro-columns, which is still 10 times higher than that of 2D planar cultures (Adewole et al., 2021). Therefore, we assume that the growth rate of cortical axons in the testbed system ranged from 0.32 ± 0.04 mm/day to 0.56 ± 0.03 mm/day as the cross-over time (3 DIV) was similar to that of our previous study. As such, the mean contraction rate (0.38 ± 0.21 mm/day) observed at 10 DIV in our system seems comparable to the growth rate considering the net length change rate of the axonal bundle.

The micro-TENNs showed maximum contraction at 7 DIV and this contraction force was enough to pull the whole beta aggregate completely toward the other end of the micro-column (Figure 3). Interestingly, we found that the aggregates showed a preference in moving towards the contraction force rather than maintaining the initial axonal length, signifying the characteristic contractile nature of these neurites. As the beta aggregate moved from one end of the micro-column towards the other end, it generated stretch-grown axons in its wake that covered more than 50% micro-column length by 10–15 DIV in some cases. Hence, we observed contraction and extension of the bundled axons in 3D due to the inherent movement of the beta aggregate in the direction of the contracted axons. The natural tendency of the axonal tract to contract post-integration implies that the cortical tissue’s innate mechanism of maintaining resting tension resulted in axonal contraction in our model.

We further wanted to visualize the neurite growth of both the alpha and beta aggregates in real time as they integrated with each other. Transduction of the neurons with different AAV vectors expressing GFP and mCherry allowed us to quantify the contraction rate and time-points of key events such as integration and peak contraction (Figure 4). Interestingly, the stretch-grown axons on the right side of the beta aggregate depicted in inset image (Figure 4C) demonstrated an overlapping of green and red channels which suggests the stretch-grown axonal tract served as pioneering axons or as a substrate for the alpha-originating axons to grow along. We found that the peak contraction occurred after the axon tracts extended fully toward the opposite aggregate, suggesting that target integration is a crucial precedent for this phenomenon. This was further corroborated by staining the constructs with synapsin-1 antibody post-contraction (Figure 5), indicating that the axons might have established putative synaptic connections with the opposite target population. However, a more in-depth investigation is needed to visualize the synaptic puncta using both pre-synaptic and post-synaptic markers and to examine the interplay between axon mechanics and synapse formation. The role of microtubules and other cytoskeletal-associated proteins in synaptogenesis is well established as the microtubule organization regulates growth cone motility and synaptic plasticity (Hummel et al., 2000; Roos et al., 2000; Miryala et al., 2022). There is increasing experimental evidence suggesting that the actomyosin contractile machinery of axons generates tension and thereby regulate synaptogenesis (Siechen et al., 2009). A study performed in the Drosophila nervous system revealed that synaptic vesicle clustering was controlled by mechanical tension within the axons. It was shown that the pre-synaptic vesicle clustering vanished upon axotomy and was restored upon applying mechanical tension to the severed end of the axon (Siechen et al., 2009). Although the study demonstrated the role of tension in neuromuscular synapses, we could assume that the cortical axons of alpha and beta aggregates in the micro-TENNs establish putative synaptic connections along the dendrites and cell bodies while integrating with each other. A significant portion of neuronal connectivity within the CNS occurs in early development and brain pathways are established according to the synapses that form upon reaching the target population (Ayali, 2010). This further provides evidence for the putative role of synapse mechanics in regulating the wiring distances and axonal contraction/stretch-growth between neuronal populations; however, more direct investigations are warranted.

We further analyzed the contraction rate of bundled axons by changing the column length and explored the mechanisms of contraction by exposing the contracted micro-TENNs to drugs known to inhibit specific cytoskeletal proteins and cytoskeletal-molecular motor interactions. Our results suggest that bundled cortical axons have an intrinsic contractile nature once integrated, which was dependent upon microtubule integrity. These results are not surprising, since cytoskeletal mechanics play a major role in maintaining homeostasis and resting tension during development (Ayali, 2010; Abuwarda and Pathak, 2020). The key players for axonal mechanics include cytoskeletal elements such as microtubules, neurofilaments, and actin (Heidemann et al., 1995). The dynamic nature of the cytoskeleton enables continuous remodeling of neural tissue and the regulation of resting tension as the neurites generate, sense, and respond to forces while growing (Kahn and Baas, 2016). Microtubule polymerization-depolymerization cycles lead to the extension and retraction of axons. This extension and contraction of axons is regulated by myriad internal and external forces recognized by the cytoskeleton (Kahn and Baas, 2016). Given this critical role of microtubule dynamics in axonal extension and retraction, it makes sense that intra-contractile forces are also dependent on microtubule dynamics and integrity.

We next wanted to begin to probe the mechanisms behind the rapid cytoskeletal change to induce contraction after target integration. We did so by inhibiting the function of proteins known to be involved in cytoskeletal remodeling; specifically, rho-kinase (a signaling molecule known to facilitate axonal guidance and regeneration), dynein (a cytoskeletal motor protein), and microtubules (the cytoskeletal backbone of axonal structure). In previous studies, axonal retraction has been tied to various structural molecules like microfilaments and microtubules, as well as motor proteins (Ahmad et al., 2000; Gallo, 2004; Mutalik et al., 2018; Guan et al., 2023). The dynamic process of axonal extension and contraction occurs in part due to microtubule polymerization and depolymerization, respectively. To reach the target tissue, axons are capable of growing to centimeter-long distances that involve stages of axonal extension and retraction in search of the target. The structural support provided by the microtubules is necessary to withstand the tension and the motor-generated forces as axons grow (Tofangchi et al., 2016). Recently, it was found that motor activity of cytoplasmic dynein protein was essential for maintaining axonal length along with microtubular activity (Ahmad et al., 2000). Rho-kinase signaling is also associated with axonal extension and neuronal migration (Fournier et al., 2003; Fujita and Yamashita, 2014). Hence, rho-kinase, dynein and microtubule stability were the main targets for our experiments, and we found significant inhibition of contraction by treating the contracted micro-TENNs with the microtubule inhibitor nocodazole (Figure 6). These results indicate that microtubule stability is at least indirectly necessary for axonal contraction, but due to the importance of microtubules for the overall structural integrity of cells, there are broad consequences to microtubule destabilization, and we therefore cannot reliably conclude that the contraction mechanism solely relies on microtubules. The rho-kinase inhibitor did not appear to inhibit contraction, indicating that despite its role in other axonal restructuring mechanisms, rho-kinase is likely not involved in promoting axonal contraction in response to target engagement. Similarly, we did not observe a significant difference in the contraction rate upon using the dynein inhibitor Ciliobrevin D; however, the treatment resulted in a trend towards reducing the rate of contraction. It might be possible that the treatment time or concentration was insufficient to produce a significant change as seen in case of microtubule destabilization. Future studies will therefore explore a wider concentration range along with prolonged treatment periods to administer the drugs, provided that such testing regimes do not affect neuronal health and viability.

The nocodazole treatment findings were noteworthy because the contraction stopped completely within the 24-h treatment. Nocodazole has been shown to inhibit axonal extension and growth in various other studies as this compound destabilizes the microtubules (Solomon and Magendantz, 1981). In fact, inhibition of microtubules with nocodazole led to axonal retraction as the neurites failed to maintain their original length (Solomon and Magendantz, 1981; Dennerll et al., 1988; Ahmad et al., 2000; Mutalik et al., 2018). Chick sensory neurons cultured on untreated glass coverslips demonstrated axonal retraction after 15 min of treatment with 10 μg/mL nocodazole (Ahmad et al., 2000). While these previous results were in different experimental paradigms, they are in contrast to our observations as we found that nocodazole prevented contraction of the bundled axons and helped maintain the original length of the integrated axonal tracts. It is important to note that unlike the previous studies, our platform resulted in axonal target integration in neuronal cell body aggregates at either end that likely anchored the axons after extension. It is possible that the retraction observed by Ahmad and colleagues was a passive process, while the contraction we observed was a force-generating active process (Ahmad et al., 2000). To our knowledge, the current study is the first report showing inhibition of contraction in 3D axonal bundles using nocodazole, highlighting the unique arrangement of this tissue-engineered *in vitro* testbed.

The contraction behavior observed in bundled cortical axons highlights the importance of such a testbed to explore neurodevelopmental mechanisms and future directions to study axonal contraction linked with brain morphogenesis. As the human brain evolved, the cerebral cortex expanded and folded many times to accommodate a large surface area, extending up to 1,600–2000 cm^2^ and fitting in a skull almost three times smaller (Sousa et al., 2017). There is evidence that the convolutions were developed as the axonal fibers attached and exerted a mechanical tension while shaping the 3D structure and developing distinct brain pathways (Hilgetag and Barbas, 2005, 2006; Sousa et al., 2017). Hilgetag and Barbas (2006) demonstrated that the tension exerted by cortico-cortical connections played a significant role in shaping the cerebral cortical landscape of the brain and the formation of gyri/sulci in the gyrencephalic cortices. This demonstrates the varying degree of mechanical forces between dense regions and weakly connected regions in the gyri and sulci respectively, disruption of which leads to developmental disorders (Clark and Plante, 1998; Hilgetag and Barbas, 2006; Bayly et al., 2013; Lin et al., 2021). Our micro-TENN testbed holds potential in understanding such neural networks by changing the neuronal populations at either side of the micro-column to study axo-dendritic, axo-somatic and axo-axonic connections. It would be interesting to see unidirectional contraction mechanism by using different types of cortical neurons from two separate regions of the brain that do not form bidirectional connections and thus do not innervate each other. One such example is the fabrication of tissue engineered nigrostriatal pathway (TE-NSP) in which the discrete populations of dopaminergic neurons and medium spiny neurons (MSNs) are connected via dopaminergic axons that innervate the MSNs and not vice versa (Struzyna et al., 2022).

The axonal tension hypothesis postulates that axons pull neural tissues as they make connections with different regions and are thus capable of forming gyri when the strongly connected areas are drawn together by the pulling axons; whereas the weakly linked areas generate sulci in the brain (Van Essen, 1997). The cortical micro-TENNs recapitulate the connections between two cortical aggregates separated within a millimeter-scale hollow hydrogel micro-column and demonstrates the physical changes that occur upon their integration. This micro-TENN testbed for axonal contraction can be instrumental in further studying axonal forces and dynamics in human cortical neurons that play a role in maintaining the foldings in cortex and the mechanism(s) behind gyri formation. Future studies in this system can continue the pharmacological interrogation of the signaling pathways and force transducers involved in axonal contraction after target integration. In addition, techniques for fabricating micro-TENNs from human stem cells could produce *in vitro* testbeds for studying axonal contraction in the context of genetic neurodevelopmental disorders. Indeed, we previously developed human stem cell-derived cortical organoid micro-TENNs to stimulate multiple cortical layers and long-distance brain pathways (Cullen et al., 2019). Such models can be further utilized to better understand axonal biomechanics. Therefore, the micro-TENN testbed holds potential for investigations of altered brain shape, structural impairments, and variations in brain connections that are associated with certain neurodevelopmental disorders. We also foresee the application of cortical micro-TENNs to model gyrencephalic brain development, as well as associated developmental disorders in the near future.

## 5 Conclusion

CNS development depends on the precise regulation of cytoskeleton organization and dynamics to attain a resting tension, thus facilitating maintenance of the structural and functional aspects of the nervous system. The study of mechanisms behind nervous system development and related disorders is still an ongoing topic of research due to the limitations in obtaining and growing human tissues. Therefore, *in vitro* tissue models are necessary to fill the gaps in our current understanding. The micro-TENN model featuring aggregated neurons and long-projecting bundled axonal tracts recapitulates key neuroanatomical features under *in vitro* conditions. We successfully designed a testbed model of cortical tissue that demonstrates cortico-cortical interactions and their inherent contractile behavior under the lack of external mechanical forces. This simple testbed demonstrated contractility of bundled axons – notably occurring after traditional growth-cone mediated axonal extension and integration – and likely represented intrinsic changes in resting tension as neural tissue grows over time.

Collectively, our data suggest that bundled axons have capacity to generate mechanical forces and are inherently contractile following target integration. These forces could be similar to the axonal tensions that drive cerebral folding or the forces responsible for minimizing the “wiring distances” between integrated brain regions, thereby shortening communication times by bringing the regions close together. Our study implicates microtubules as necessary for axonal contractility once the two neuronal regions are interconnected. In future applications, this biofidelic testbed may be used to model structural aspects of human CNS development to improve our understanding of brain morphogenesis and mechanisms underlying certain neurodevelopmental disorders.

## Data availability statement

The raw data supporting the conclusions of this article will be made available by the authors, without undue reservation.

## Ethics statement

The animal study was approved by Institutional Animal Care and Use Committee (IACUC) by the University of Pennsylvania and the Corporal Michael J. Crescenz Veterans Affairs Medical Center. The study was conducted in accordance with the local legislation and institutional requirements.

## Author contributions

DC: Conceptualization, Data curation, Formal analysis, Methodology, Validation, Visualization, Writing – original draft. WGV: Conceptualization, Data curation, Formal analysis, Methodology, Validation, Visualization, Writing – review & editing. LAS: Data curation, Methodology, Writing – review & editing. DOA: Data curation, Methodology, Writing – review & editing. ERC: Writing – review & editing, Data curation. JCB: Methodology, Writing – review & editing, Data curation. JO’D: Conceptualization, Data curation, Formal analysis, Investigation, Methodology, Validation, Visualization, Writing – review & editing. DKC: Conceptualization, Formal analysis, Funding acquisition, Investigation, Methodology, Project administration, Resources, Software, Supervision, Validation, Visualization, Writing – review & editing.
