# Supplementary material for: Generation of contractile forces by three-dimensional bundled axonal tracts in micro-tissue engineered neural networks
Source: Front Mol Neurosci. 2024 Mar 25;17:1346696. doi: 10.3389/fnmol.2024.1346696 (PMC10999686; doi:10.3389/fnmol.2024.1346696)
Supplement: Supplementary file 1 [file Data_Sheet_1.docx]

Supplementary Material

**Generation of Contractile Forces By Three-Dimensional Bundled**

**Axonal Tracts in Micro-Tissue Engineered Neural Networks**

Dimple Chouhan^†1,2^, Wisberty J. Gordián Vélez^†1,2,3^, Laura A. Struzyna^1,2,3^, Dayo O. Adewole^1,2,3^, Erin R. Cullen^1,2^, Justin C. Burrell^1,2^, John C. O’Donnell^1,2,3^, D. Kacy Cullen^1,2,3^*

(1) Center for Brain Injury & Repair, Department of Neurosurgery, Perelman School of Medicine, University of Pennsylvania, Philadelphia, PA;

(2) Center for Neurotrauma, Neurodegeneration & Restoration,

Michael J. Crescenz Veterans Affairs Medical Center, Philadelphia, PA;

(3) Department of Bioengineering, School of Engineering and Applied Science,

University of Pennsylvania, Philadelphia PA;

**†These authors contributed equally to this work and share first authorship**

***Frontiers in Molecular Neuroscience***

*Special Research Topic: Perspectives in Neuroscience: Mechanical Forces in the Modulation of Axonal Mechanics and Nerve Regeneration*

Corresponding author:

D. Kacy Cullen, Ph.D.

105E Hayden Hall/3320 Smith Walk

Dept. of Neurosurgery

University of Pennsylvania

Philadelphia, PA 19104

Ph: 215-746-8176

Fx: 215-573-3808

Email: [dkacy@mail.med.upenn.edu](mailto:dkacy@mail.med.upenn.edu)

Running title: Axon Contraction in Micro-Tissue Engineered Neural Networks

Keywords: axon tracts, mechanical forces, axon contraction, axon mechanics, cortical neurons

*
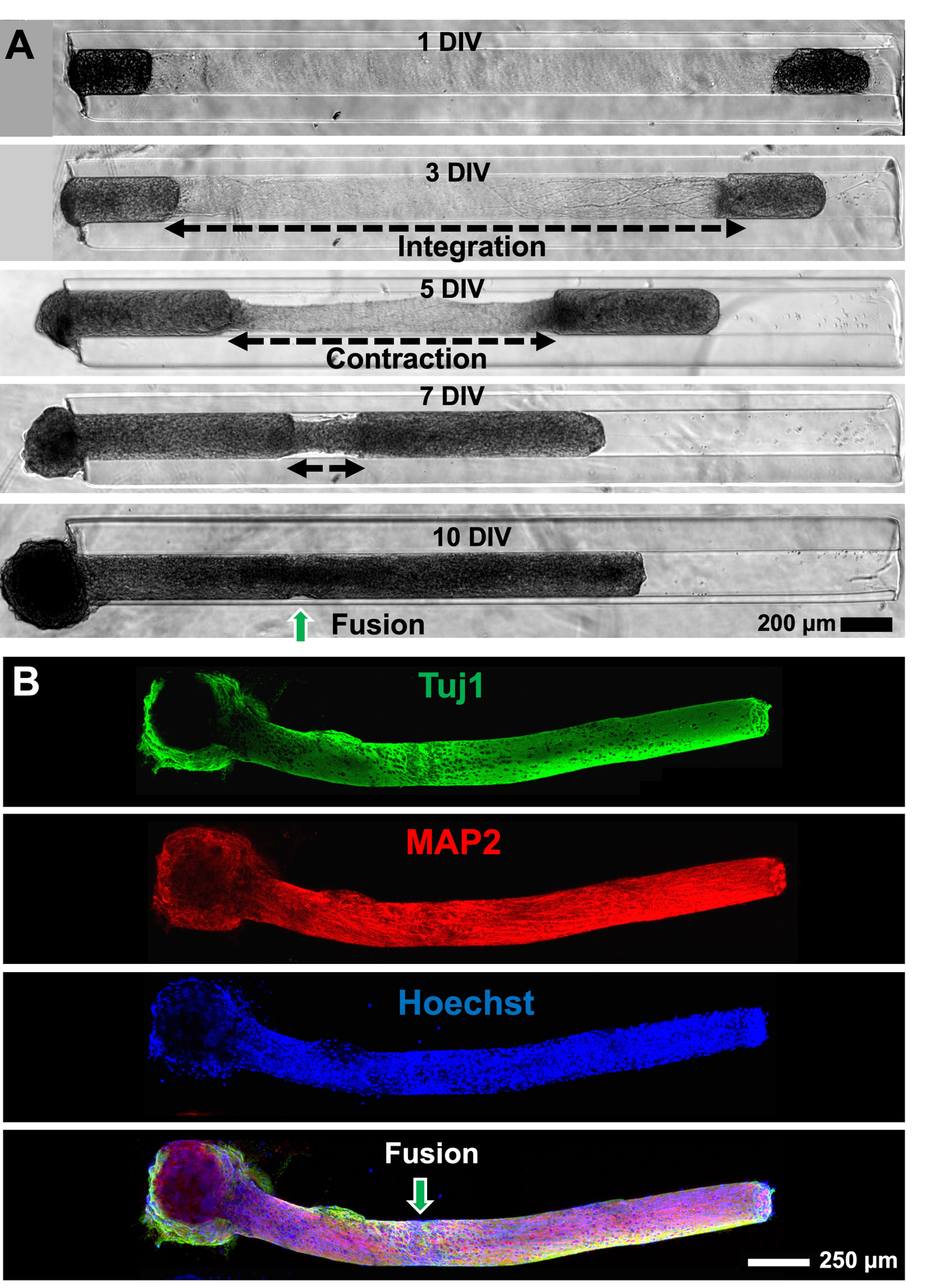
*

**Supplementary Figure 1.** An example of axonal contraction leading to complete aggregate fusion in a micro-TENN with little to no visible axonal bundle between the two aggregates at 10 DIV. (A) Phase contrast images of the construct as the tissue grows over time from 1 to 10 DIV. (B) Confocal reconstructions of the micro-TENN fixed at 10 DIV validate complete fusion of regions depicted by the presence of nuclear marker (Hoechst; blue) throughout the length of the construct and absence of a distinct axonal region and stretch-grown axons. Scale Bars: A: 200 µm, B: 250 µm.

**
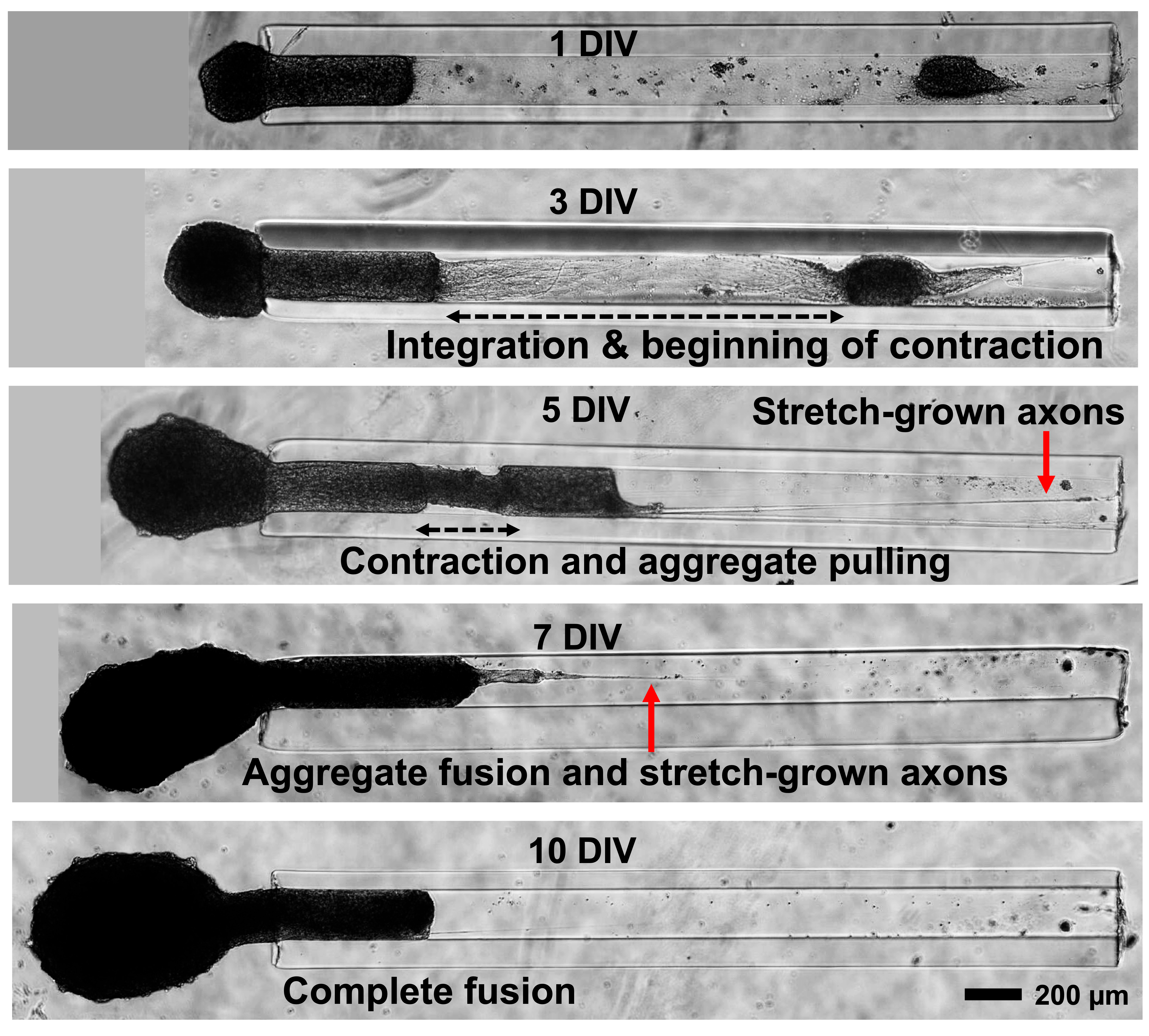
**

**Supplementary Figure 2.** Another example of axonal contraction leading to complete aggregate fusion in a micro-TENN. Stretch-grown axons (red arrows) are also apparent at 5 DIV and 7 DIV. The phase contrast images highlight complete fusion of two aggregates at later time-points with little to no visible axonal bundle between the two aggregates. Scale Bar: 200 µm.

*
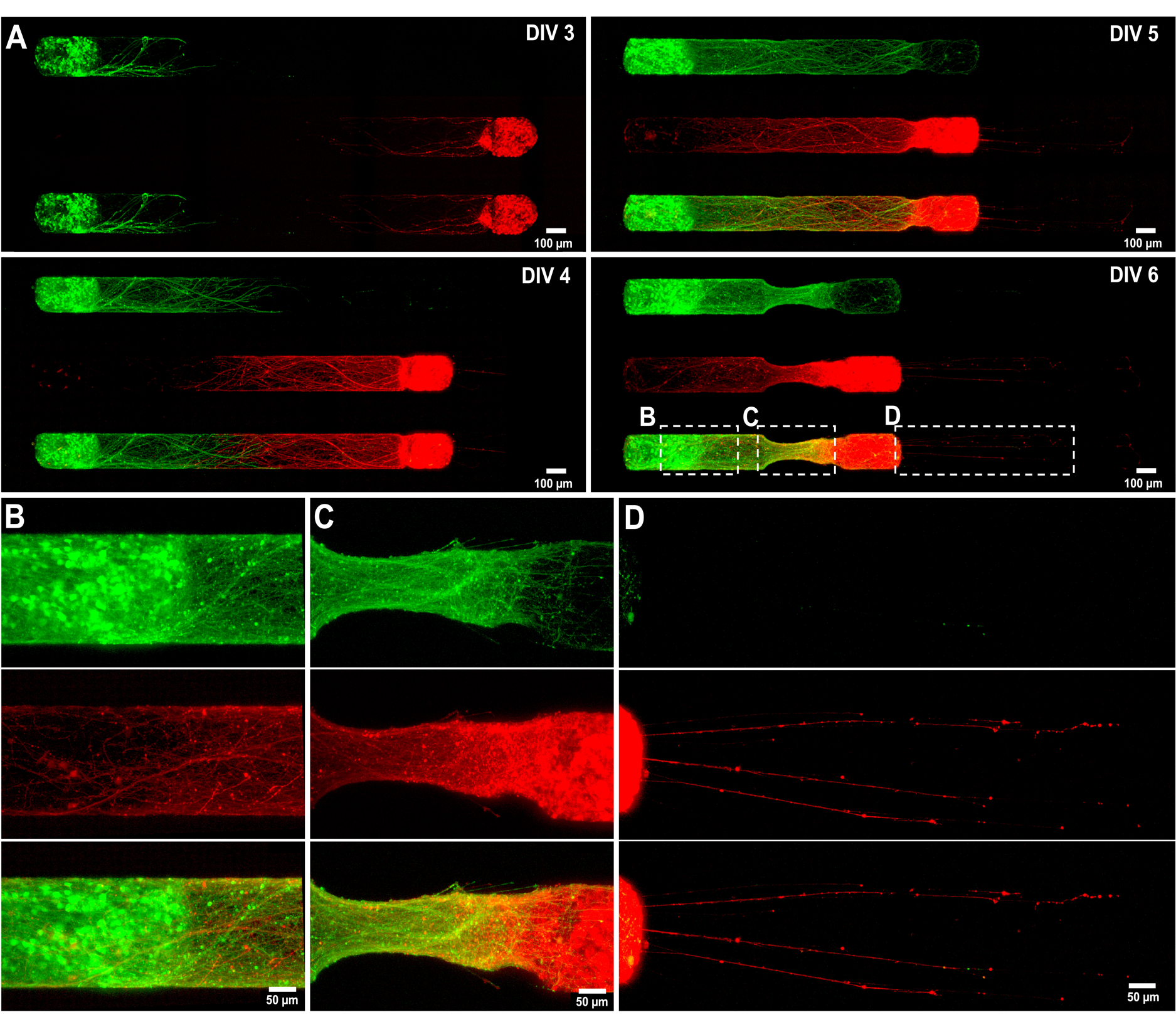
*

**Supplementary Figure 3.** An example of GFP-mCherry labelled aggregates in the micro-TENN as the neural tissue develops over time. (A) Confocal 3D reconstructions depicting live images of the growing construct over time to show the changes occurring as the tissue matures. (B-D) The inset images display zoom-in regions of the GFP+ aggregate, integrated contracted axonal bundle near mCherry+ aggregate and stretch-grown axons post-contraction at 6 DIV. Herein, GFP+ and mCherry+ axons were observed in the opposite aggregate showing innervation into each other; however, an overlay of GFP and mCherry in the stretch-grown axons was not found suggesting stretch-growth of only the mCherry+ aggregate. Scale Bars: A: 100 µm, B-D: 50 µm.

*
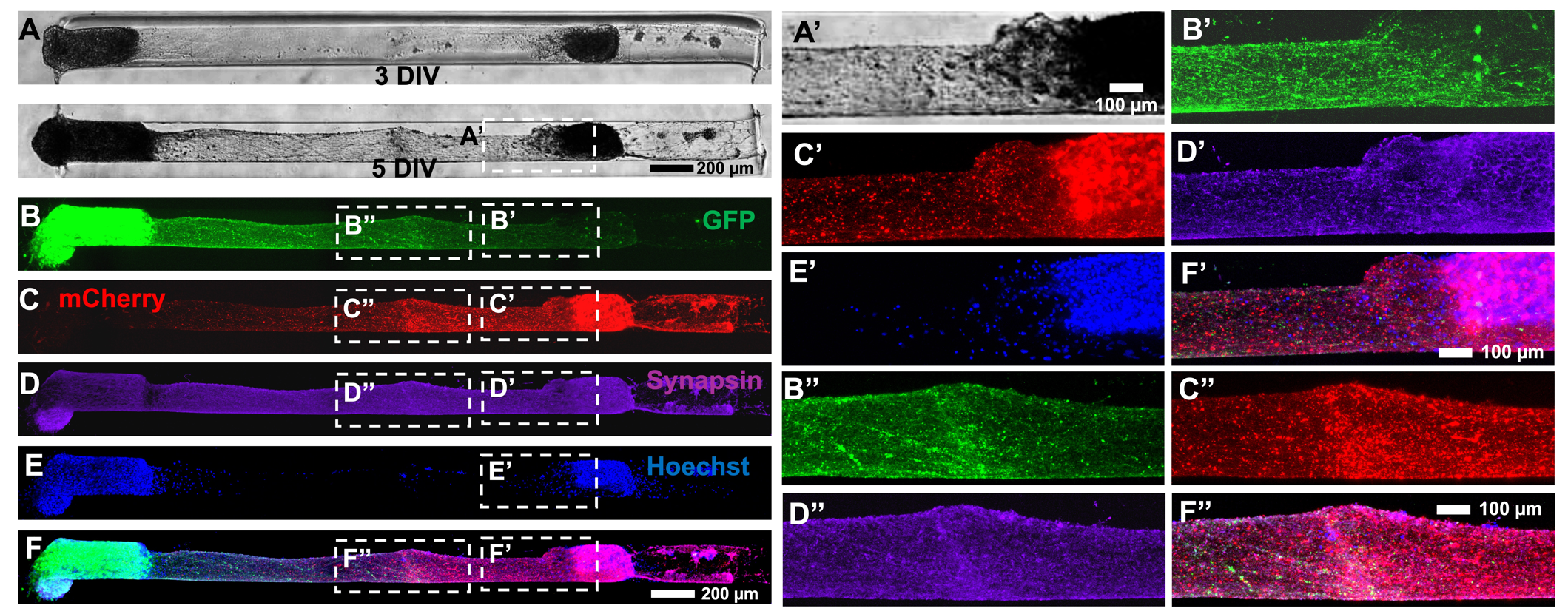
*

**Supplementary Figure 4. Synaptic integration in cortical micro-TENNs.** Micro-TENN cytoarchitecture was observed by immunolabelling the micro-TENNs at 5 DIV. (A) Phase contrast images of the representative construct at 3 and 5 DIV are shown prior to fixing the constructs. The constructs were fixed at 5 DIV as integration of the axonal bundles was first observed at this time-point. (B-F) Cells in the micro-TENN transduced with AAV vectors expressed GFP and mCherry; they were further stained for presynaptic marker – synapsin-1 (far red) and nuclei (Hoechst; blue) to demonstrate the presence of pre-synaptic terminal protein Synapsin-I in the neural tissue as the axons integrated and began to contract. Insets highlighted by the white dashed boxes refer to the zoom-in images in individual channels. Confocal reconstructions show labelled cell-aggregate region (B’-F’), part of the bundled axonal region (B’’-F’’). Scale bars: A-F: 200 µm, A’-F’’: 100 μm.

**
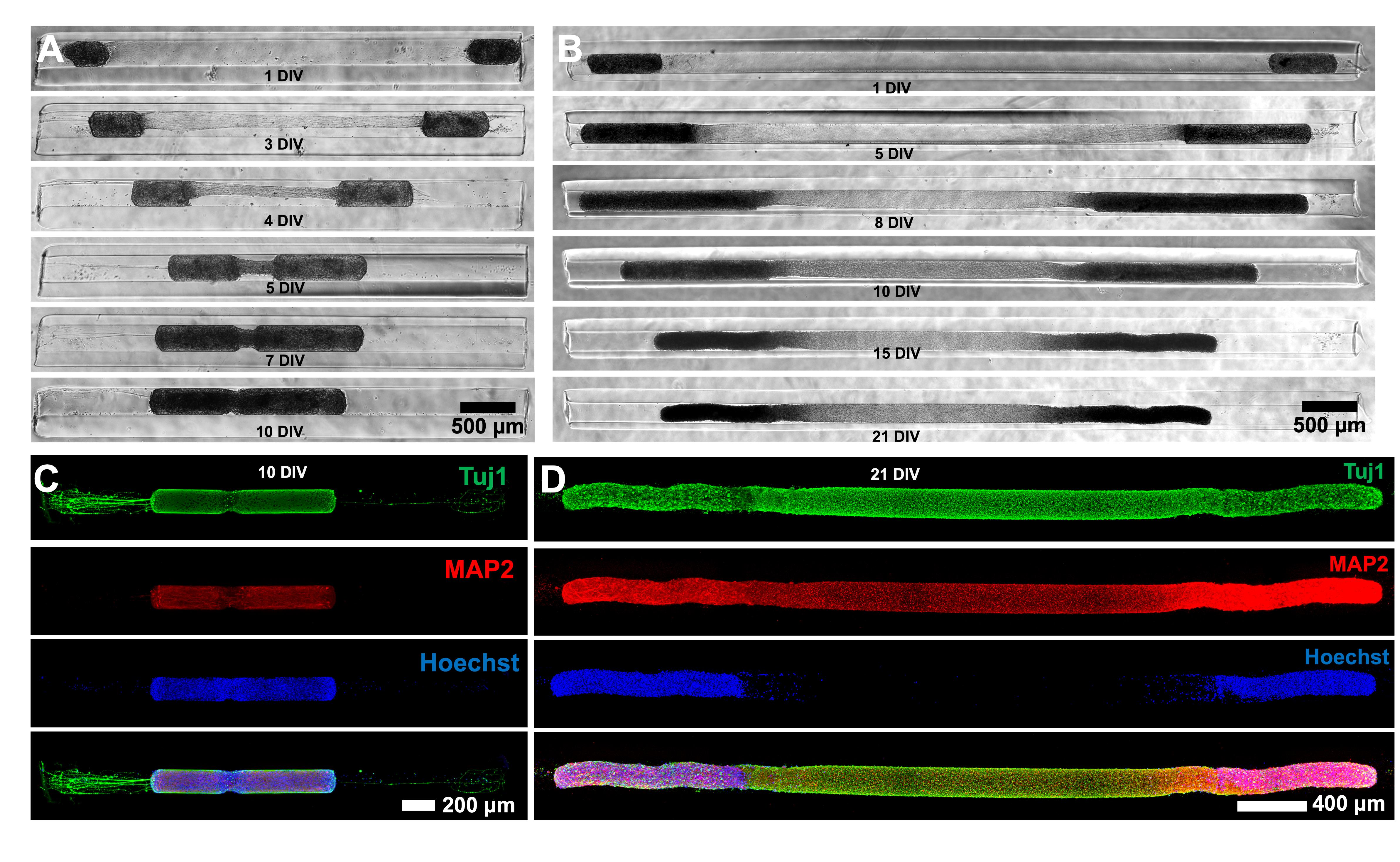
Supplementary Figure 5.** An example of comparison between 3 mm and 7 mm long micro-TENNs showing homogenous contraction from both sides. Both the types of constructs were developed using equal sized aggregates on each side. (A) Representative phase contrast micrographs of a 3 mm long micro-TENN at various time-points showing contraction of the bundled axons between the two aggregates. (B) Representative phase contrast images of a 7 mm long construct at various time-points showing presence of intact bundled axonal tract between the two aggregates. (C and D) The ICC images represent the characteristic markers of cortical neurons stained using anti-Tuj1 (green; all axons), anti-MAP2 (red; soma) and Hoechst nuclear marker (blue). Micro-columns of different lengths show that axonal contraction is influenced by the distance between the two aggregates. Scale Bars: A, B: 500 µm, C: 200 µm, D: 400 µm.

*
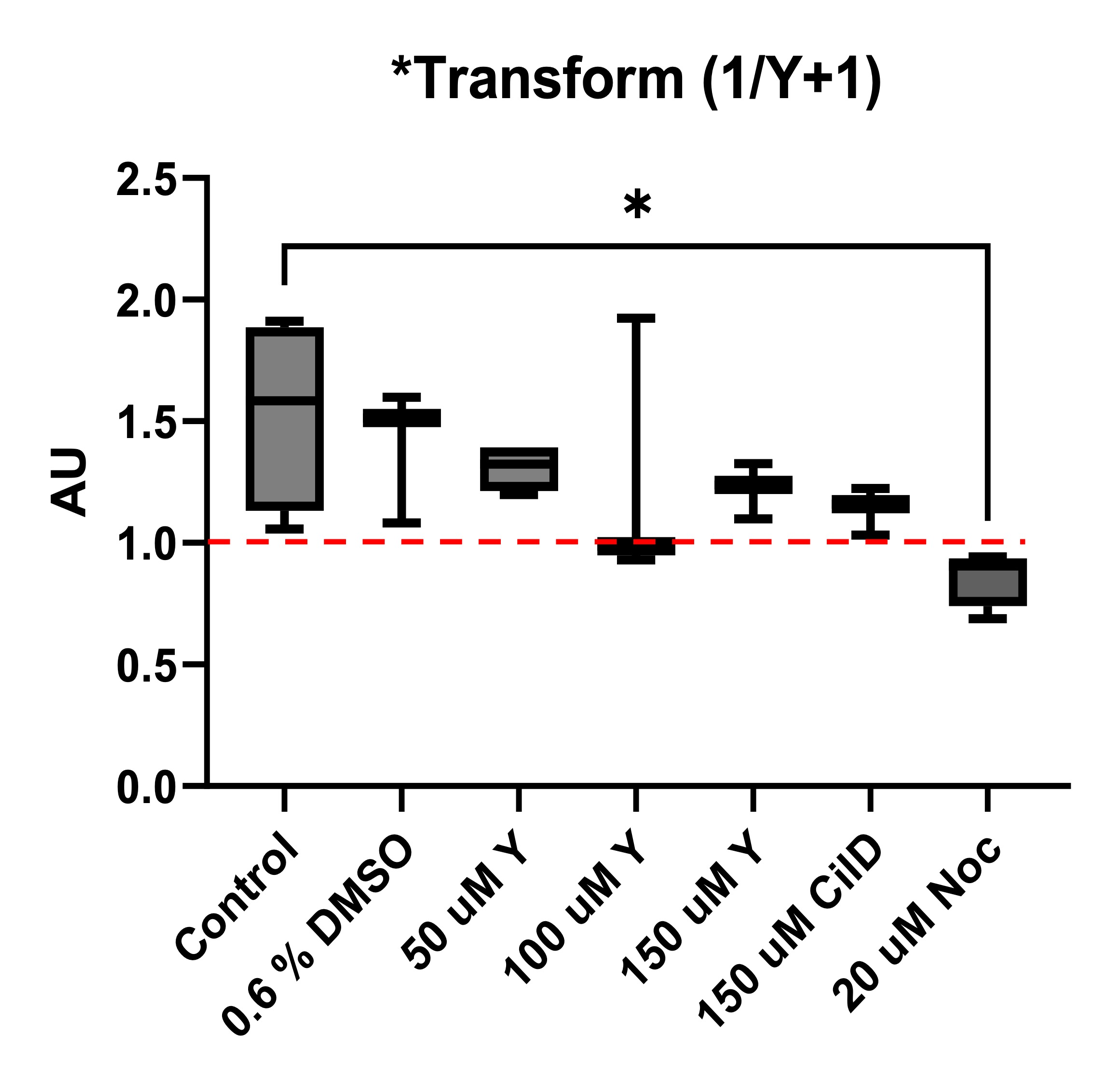
*

**Supplementary Figure 6.** Box plot graph showing the values obtained after transforming the data shown in Figure 6H to satisfy assumptions of normality for ANOVA.
